# *TP53* loss initiates chromosomal instability in fallopian tube epithelial cells

**DOI:** 10.1242/dmm.049001

**Published:** 2021-11-30

**Authors:** Daniel Bronder, Anthony Tighe, Darawalee Wangsa, Dali Zong, Thomas J. Meyer, René Wardenaar, Paul Minshall, Daniela Hirsch, Kerstin Heselmeyer-Haddad, Louisa Nelson, Diana Spierings, Joanne C. McGrail, Maggie Cam, André Nussenzweig, Floris Foijer, Thomas Ried, Stephen S. Taylor

**Affiliations:** 1 Division of Cancer Sciences, Faculty of Biology, Medicine and Health, University of Manchester, Manchester Cancer Research Centre, Wilmslow Road, Manchester M20 4GJ, UK; 2 Genetics Branch, National Cancer Institute, National Institutes of Health, Bethesda, MD 20892, USA; 3 Laboratory of Genome Integrity, National Cancer Institute, National Institutes of Health, Bethesda, MD 20892, USA; 4 CCR Collaborative Bioinformatics Resource, Center for Cancer Research, National Cancer Institute, National Institutes of Health, Bethesda, MD 20892, USA; 5 European Research Institute for the Biology of Ageing (ERIBA), University of Groningen, University Medical Center Groningen, 9713 AV Groningen, The Netherlands

**Keywords:** High-grade serous ovarian cancer, Fallopian tube, Chromosomal instability, *TP53*, *BRCA1*, *MYC*

## Abstract

High-grade serous ovarian cancer (HGSOC) originates in the fallopian tube epithelium and is characterized by ubiquitous *TP53* mutation and extensive chromosomal instability (CIN). However, direct causes of CIN, such as mutations in DNA replication and mitosis genes, are rare in HGSOC. We therefore asked whether oncogenic mutations that are common in HGSOC can indirectly drive CIN in non-transformed human fallopian tube epithelial cells. To model homologous recombination deficient HGSOC, we sequentially mutated *TP53* and *BRCA1* then overexpressed *MYC*. Loss of p53 function alone was sufficient to drive the emergence of subclonal karyotype alterations. *TP53* mutation also led to global gene expression changes, influencing modules involved in cell cycle commitment, DNA replication, G2/M checkpoint control and mitotic spindle function. Both transcriptional deregulation and karyotype diversity were exacerbated by loss of *BRCA1* function, with whole-genome doubling events observed in independent p53/BRCA1-deficient lineages. Thus, our observations indicate that loss of the key tumour suppressor *TP53* is sufficient to deregulate multiple cell cycle control networks and thereby initiate CIN in pre-malignant fallopian tube epithelial cells.

This article has an associated First Person interview with the first author of the paper.

## Abstract (precis)

**Summary:** High-grade serous ovarian cancer is defined by *TP53* mutation and chromosomal instability (CIN), the cause of which remains poorly understood. We developed a novel model system that implicates cell cycle deregulation upon p53 loss as cause of CIN.

## INTRODUCTION

High-grade serous ovarian cancer (HGSOC) is the most common histological subtype of ovarian cancer and the deadliest gynaecological malignancy (Bowtell et al., 2015). Survival statistics are dismal, with 5-year survival of ∼30%, and have remained largely unchanged for 30 years, illustrating the need for improved therapeutic interventions, requiring a better understanding of underlying disease biology.

HGSOC is characterized by a relatively low mutational burden at the nucleotide level (Ciriello et al., 2013). *TP53* mutations, which are present in precursor lesions, are ubiquitous and considered an early, truncal event in HGSOC tumorigenesis (Ahmed et al., 2010; Labidi-Galy et al., 2017; Vang et al., 2016). However, with the exception of *BRCA1/2* mutations in ∼25% of cases, other common driver mutations are rare (Cancer Genome Atlas Research Network, 2011). By contrast, HGSOC genomes are characterized by extensive chromosomal copy number aberrations, a consequence of rampant chromosomal instability (CIN) (Cancer Genome Atlas Research Network, 2011; Nelson et al., 2020). Indeed, HGSOC ranks among the most chromosomally unstable tumour types (Ciriello et al., 2013; Shukla et al., 2020), a characteristic confirmed by recent live-cell imaging of established cell lines and patient-derived *ex vivo* cultures, which revealed an unprecedented level of chromosome segregation errors (Nelson et al., 2020; Tamura et al., 2020).

To delineate the mechanisms responsible for CIN, HGSOC genomes have been extensively studied by whole-genome sequencing, with one study defining two CIN classes, characterized either by homologous recombination deficiency (HRD) or foldback inversions (FBI) (Wang et al., 2017). Whereas the former correlated with mutations in *BRCA1/2*, amplifications of *MECOM* and *MYC*, and loss of *RB1*, the latter correlated with *CCNE1* amplification and *PTEN* loss (Wang et al., 2017). A second study identified seven CIN signatures, including whole-genome duplication (WGD), suggesting a larger array of underlying driver mechanisms in addition to HRD and FBI (Macintyre et al., 2018).

This presents a paradox; although HGSOC appears to be driven by CIN, mutations in genes ensuring faithful cell division and DNA replication are extremely rare (Bastians, 2015). HRD, either as a consequence of *BRCA1/2* inactivation or mutation in other DNA damage repair genes, is an obvious contributor to CIN, but by itself can only account for up to ∼50% of cases (Cancer Genome Atlas Research Network, 2011; Weaver et al., 2002; Xu et al., 1999). Aside from homologous recombination, an additional role for BRCA1 in maintaining chromosomal stability has also been described (Di Paolo et al., 2014; Yu et al., 2012). *TP53* mutations have consistently been shown to correlate with aneuploidy (Ciriello et al., 2013; Davoli et al., 2017; Taylor et al., 2018; Zack et al., 2013), but the role of *TP53* as a driver of CIN remains controversial. Initial studies using the near-diploid colorectal cancer cell line HCT116 suggested that p53 (encoded by *TP53*) loss is not sufficient to cause CIN (Bunz et al., 2002). However, suppressing p53 in *hTERT*-immortalized RPE-1 cells, or in diploid and tetraploid murine mammary epithelial cells, did generate abnormal karyotypes (Fujiwara et al., 2005; Kok et al., 2020; Soto et al., 2017). Furthermore, p53 inactivation in transformed murine embryonic fibroblasts deregulated multiple cellular processes affecting DNA damage response, mitosis and ploidy control (Valente et al., 2020).

Here, we aimed to develop novel model systems of CIN in HGSOC, starting with *hTERT*-immortalized non-ciliated fallopian tube epithelial cells (Merritt et al., 2013). In the first instance, we set out to model the HRD CIN class, using CRISPR/Cas9-mediated gene editing to first mutate *TP53* then *BRCA1*, followed by overexpression of *MYC.* A panel of derivative subclones was subjected to functional assays, karyotyping and transcriptional profiling to determine (1) whether CIN had been induced and (2) what the potential mechanisms might be.

## RESULTS

### FNE1 cells to model CIN in HGSOC

**Fig. 1.**
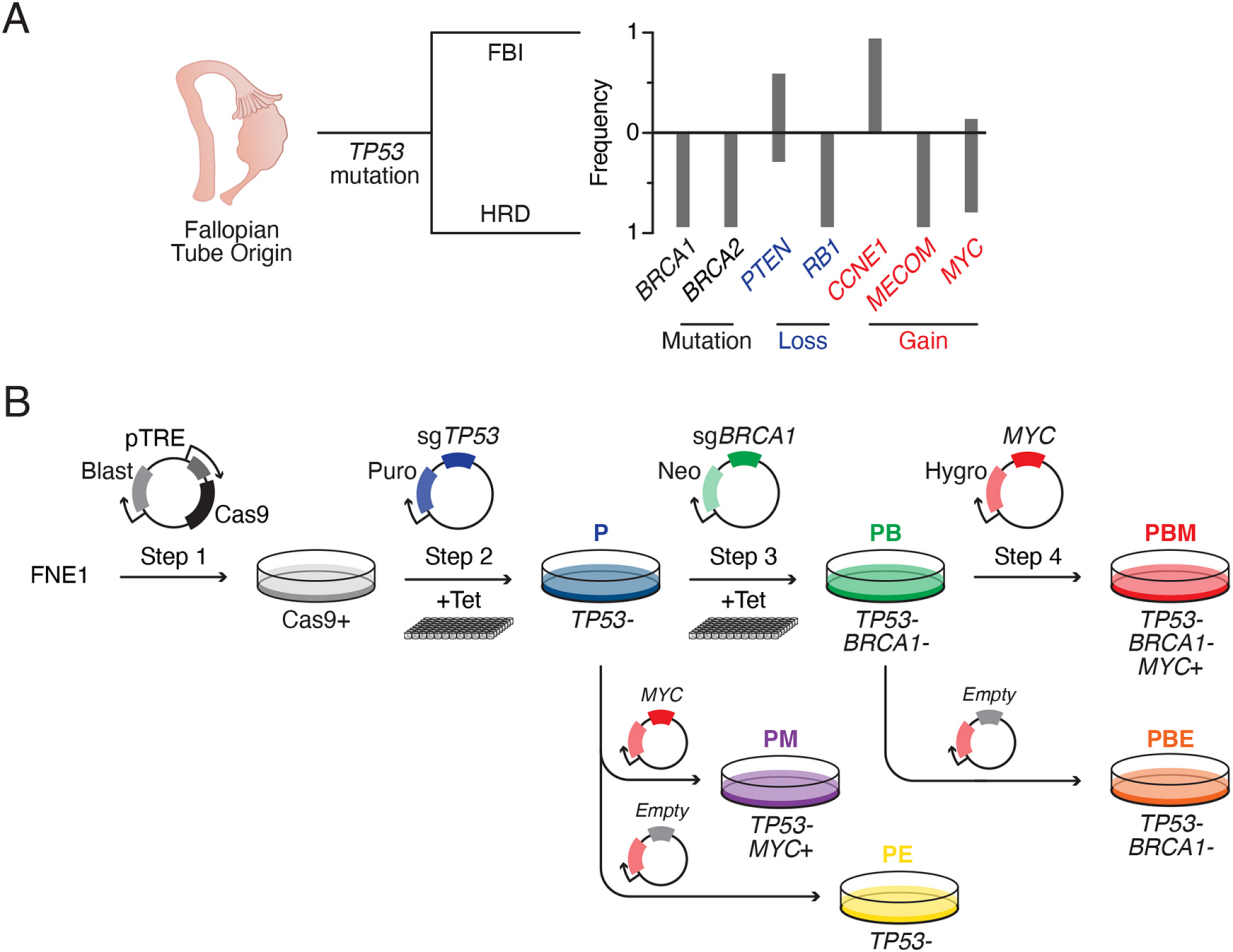
**Intellectual framework and experimental strategy.** (A) Schematic of modelled high-grade serous ovarian cancer (HGSOC) development from the fallopian tube secretory epithelium, including ubiquitous *TP53* mutation, grouping based on foldback inversions (FBI) or homologous recombination deficiency (HRD) and associated genomic changes in key tumour suppressors and oncogenes (Wang et al., 2017). (B) Experimental approach using *hTERT*-immortalized, fallopian tube-derived FNE1 cells to generate tetracycline (Tet)-inducible Cas9-expressing cells, which were then mutagenized to generate isogenic p53-deficient (P), p53/BRCA1-deficient (PB) and MYC-overexpressing double (PM)- and triple (PBM)-mutant subclones. MYC-overexpressing cells are co-isogenic, polyclonal populations of the parental subclones. Single (PE)- and double (PBE)-mutant control cells were also generated via transduction with an ‘empty-vector’ control virus. See Fig. S2A. Blast, blasticidin S; Hygro, hygromycin; Neo, neomycin; Puro, puromycin.

In addition to the truncal *TP53* mutation, *BRCA1/2* mutations and *MYC* overexpression tend to co-occur (Wang et al., 2017), suggesting that HRD and oncogene hyperactivation likely facilitate CIN development in HGSOC (Fig. 1A). To model these events, we set out to manipulate diploid, karyotypically stable cells, sequentially mutating *TP53* and *BRCA1* using CRISPR/Cas9-mediated gene editing, followed by ectopic overexpression of *MYC* (Fig. 1B). Because the fallopian tube epithelium is the likely origin for HGSOC, we chose the human FNE1 cell line (Ducie et al., 2017; Merritt et al., 2013). FNE1 is derived from non-ciliated fallopian tube epithelial cells and immortalized by ectopic expression of the telomerase component *hTERT* (Merritt et al., 2013). Importantly, FNE1 cells are *TP53* proficient, evidenced by nuclear accumulation of p53 and p21 (encoded by *CDKN1A*) induction in response to the MDM2 inhibitor Nutlin-3 (Fig. S1A,B) (Vassilev et al., 2004). In addition, FNE1 cells are near diploid and karyotypically stable, as confirmed by single-cell whole-genome sequencing (scWGS) and spectral karyotyping (SKY). scWGS showed that the genome is largely disomic, except for monosomies at 9p, 15 and X (Fig. S1C). Consistently, SKY showed a clonal loss of chromosomes 15 and X and an unbalanced translocation between the short arm of chromosome 9 and chromosome 15 (Fig. S1D). An identical FNE1 karyotype was also recently reported using multiplex fluorescence *in situ* hybridization (M-FISH) (Tamura et al., 2020). To enable CRISPR/Cas9-mediated gene editing, we transduced FNE1 cells with a lentivirus expressing a tetracycline-inducible Cas9 transgene. Increasing concentrations of tetracycline resulted in a dose-dependent induction of Cas9 (Fig. S1E). Importantly, in the absence of tetracycline, Cas9 was undetectable, thereby minimizing exposure of the genome to endonuclease activity during routine cell culture.

### CRISPR/Cas9-mediated mutation of *TP53* and *BRCA1*

**Fig. 2.**
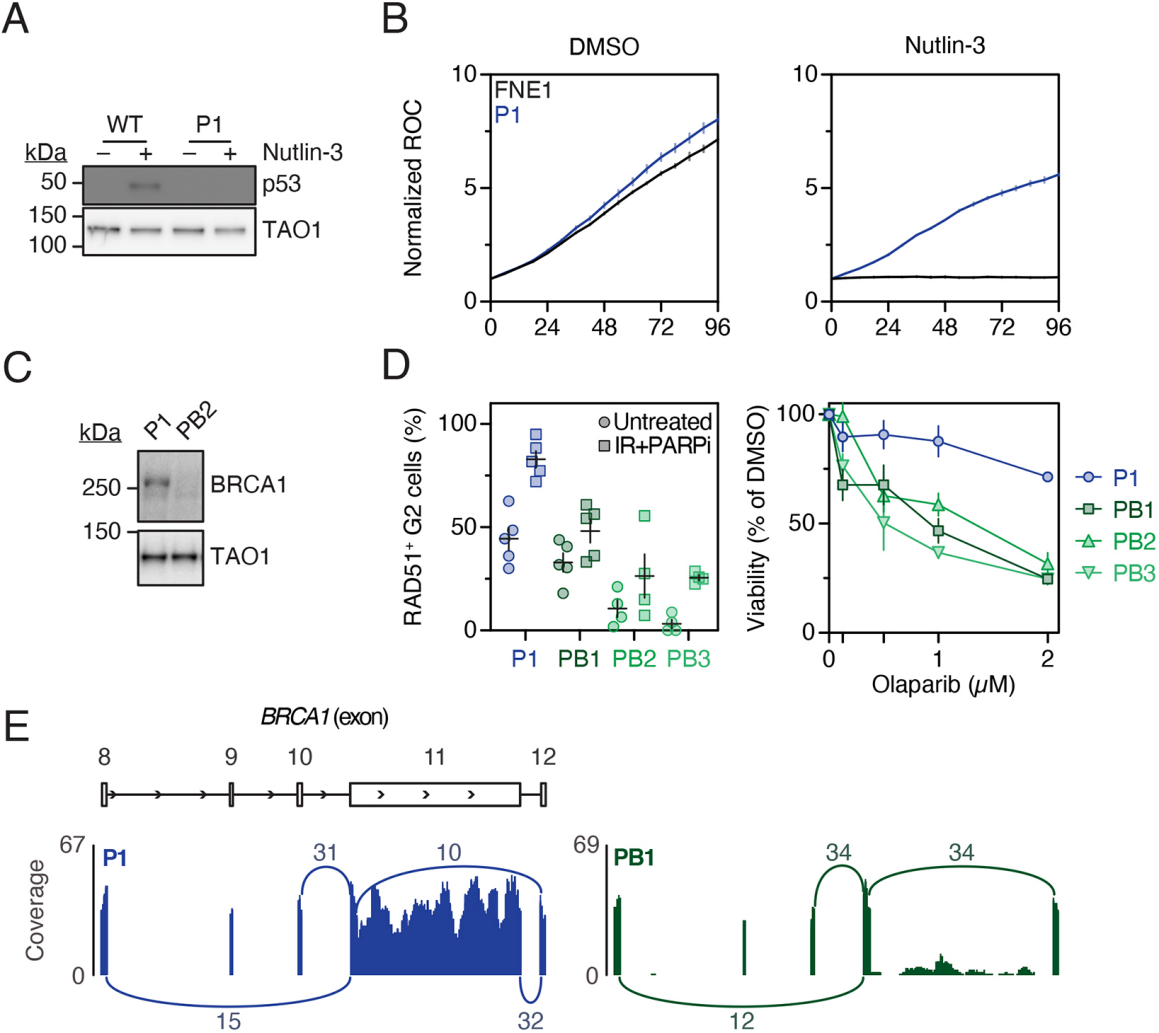
**Generation and functional validation of *TP53* and *TP53*/*BRCA1*-mutant subclones.** (A) Representative immunoblot of p53 expression in CRISPR/Cas9-derived *TP53*-mutant (P1) cells and parental FNE1 cells treated with either DMSO (vehicle) or Nutlin-3. TAO1 serves as loading control. (B) Nuclear proliferation curves of parental FNE1 and P1 cells expressing an mCherry-tagged histone in the presence of DMSO (left) or Nutlin-3 (right). Normalized red object count (ROC) was calculated as fold change from *t*_0_. Results are mean from three technical replicates with error bars indicating s.d. (C) Representative immunoblot of full-length BRCA1 expression in CRISPR/Cas9-derived *TP53/BRCA1* double-mutant (PB2) cells. Here, P1 reflects a BRCA1-proficient (p53-deficient) subclone recovered after Cas9 induction. TAO1 serves as loading control. (D) Left: quantitation of RAD51-positive G2 cells 24 h after exposure to 2 Gy X-ray and 1 μM PARPi Olaparib. Results are from at least three independent experiments; error bars represent s.e.m. Note that these data are reproduced in Fig. S3C. Right: CellTiter-Blue^®^ viability assay of P1 and PB1–3 cells treated with indicated concentrations of the PARPi Olaparib over the course of 1 week. Viability was normalized to DMSO (vehicle)-treated cells. Results are from three technical replicates, error bars represent s.d. (E) Representative Sashimi plot depicting alternative splicing of *BRCA1* exon 11 observed in P1 and PB1 subclones. Numbers indicate raw junction reads attesting to the splice events indicated by the arcs. The minimum of splice junction reads was 3. Note that junction reads mapping 3′ terminally of exon 11 and 5′ terminally of exon 12 in PB1 are not detected in PB1. P, *TP53* mutant; B, *BRCA1* mutant. See also Figs S1–S3 and Table 1.

To mutate *TP53*, we introduced a single-guide RNA (sgRNA) targeting exon 2, induced Cas9 then isolated subclones by limiting dilution, either with or without Nutlin-3 selection (Fig. 1B). Characterization of three independent subclones, designated P1–3 (Table 1; Fig. S2A), showed an absence of p53 protein (Fig. 2A), and interrogation of RNA sequencing (RNAseq) data showed that all three clones harboured frameshift mutations leading to premature termination codons (Table 1; Fig. S2B). Importantly, Nutlin-3 did not exert an anti-proliferative effect in the *TP53* mutants (Fig. 2B), indicating that the subclones are indeed functionally p53 deficient.

**Table 1.**
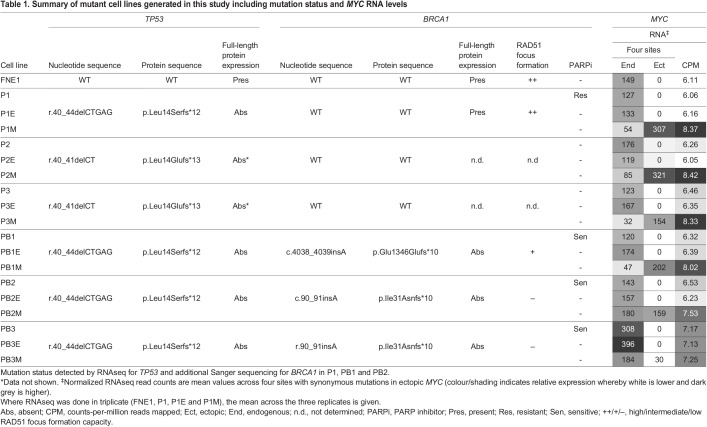
Summary of mutant cell lines generated in this study including mutation status and *MYC* RNA levels

To then mutate *BRCA1*, clone P1 was transduced with sgRNAs targeting exons 2, 3 and 11, Cas9 induced and subclones isolated by limiting dilution (Fig. 1B). Again, we characterized three independent subclones, designated PB1–3 (Table 1; Fig. S2A). Consistent with *BRCA1* mutation, immunoblotting failed to detect full-length protein, mitotic aberrations were observed in PB2 and PB3 in unperturbed conditions, induction of RAD51 foci in response to ionizing radiation was suppressed, and sensitivity to the PARP inhibitor (PARPi) Olaparib was increased (Fig. 2C,D; Fig. S3A–C). Of note, RAD51 focus formation was suppressed to a similar extent in PB2 and PB3 cells, but to a lesser extent in PB1 cells. To define the nature of the *BRCA1* mutations, we interrogated RNAseq data and mutations identified were then confirmed by Sanger sequencing of cloned genomic DNA (Table 1). PB2 and PB3 harboured mutations in exon 3, while PB1 harboured a mutation in exon 11. Interestingly, we observed alternative splicing of exon 11 in PB1 (Fig. 2E) and the expression of a truncated BRCA1 protein (Fig. S3D). Thus, although all three PB subclones harbour BRCA1 mutations, PB1 appears to retain partial BRCA1 function. Altogether, these observations confirm successful generation of FNE1 subclones harbouring mutations in both *TP53* and *BRCA1*.

### Ectopic overexpression of *MYC*

**Fig. 3.**
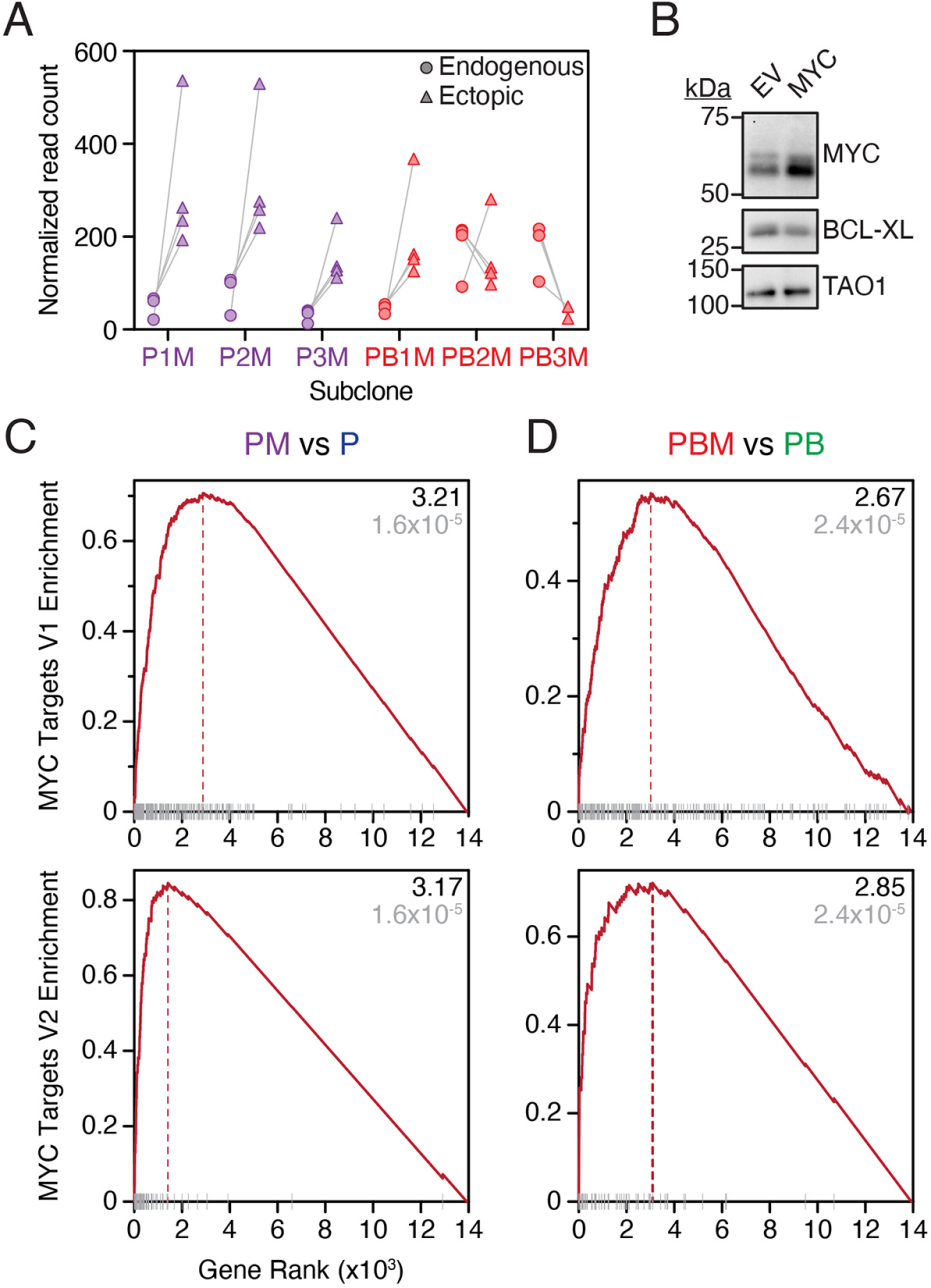
**Generation and functional validation of *MYC*-overexpressing *TP53*-mutant and *TP53/BRCA1*-mutant subclones.** (A) Normalized read count of endogenous (circles) and ectopic (triangles) *MYC* RNA was determined by interrogating RNAseq at the nucleotide level. Read counts at four sites of synonymous mutations in ectopic *MYC* were enumerated, with each mutation site reflected by one of the four circles/triangles per cell line. Reads were normalized to uniquely mapped reads. P1M was sequenced in triplicate; thus, the average of the three replicates is plotted for each locus. Note that endogenous *MYC* levels may be elevated in PB2M and PB3M relative to other samples (see Results section). (B) Representative immunoblot of P1 cells transduced with empty-vector (EV) or MYC-overexpressing (MYC) lentiviruses showing MYC and BCL-XL expression. TAO1 serves as loading control. (C,D) Enrichment of Hallmark MYC targets V1 (top) and V2 (bottom) comparing PM (pooled P1–3M) with P (pooled P1–3 and P1–3E) (C) and PBM (pooled PB1–3M) with PB (pooled PB1–3 and PB1–3E) (D). Black font indicates normalized enrichment score, and grey font indicates adjusted *P*-value. The adjusted *P*-values for differentially expressed genes in C and D were determined using the Benjamini–Hochberg algorithm. Results are from a single experiment with pooled clones as described (except for FNE1, P1, P1E and P1M, for which three technical replicates are included). P, *TP53* mutant; B, *BRCA1* mutant; M, *MYC*-overexpressing lentivirus. See Fig. S2 and Table 1.

Following mutation of *TP53* and *BRCA1*, we set out to overexpress *MYC*, an oncogene frequently amplified in HGSOC. Indeed, across 18 tumour types, HGSOC displays the highest frequency of *MYC* amplification (Zeng et al., 2018). The three *TP53*-mutant clones, P1–3, and the three P1-derived *TP53*/*BRCA1* double-mutant clones, PB1–3, were all transduced with a lentivirus harbouring a *MYC* complementary DNA (cDNA) downstream of a constitutive cytomegalovirus (CMV) promoter, generating six polyclonal derivatives, designated P1–3M and PB1–3M (Fig. 1B; Fig. S2A). In parallel, we transduced an ‘empty-vector’ control virus, generating a further six polyclonal derivatives, designated P1–3E and PB1–3E. Note that the *MYC* cDNA harboured four synonymous mutations (Littler et al., 2019), allowing differentiation of ectopic and endogenous *MYC* transcripts. In turn, RNAseq revealed that ectopic *MYC* was indeed overexpressed relative to endogenous *MYC* in P1–3M and PB1M (Fig. 3A). In PB2M and PB3M, however, the situation was reversed, possibly indicating that endogenous *MYC* was already overexpressed in these two lineages. Indeed, *MYC* was highly expressed in PB3 and PB3E, consistent with spontaneous upregulation prior to our efforts to experimentally overexpress *MYC* (Table 1). However, for the PB2 lineage, *MYC* levels were only elevated in PB2M as expected following ectopic *MYC* overexpression, and not in PB2 or PB2E.

Importantly, overexpression of *MYC* modulated MYC-dependent processes, evidenced by immunoblotting of P1M cells, which revealed downregulation of the pro-survival factor BCL-XL (Fig. 3B). Consistent with the transcriptional activity of MYC, gene set enrichment analysis (GSEA) showed that *MYC* Hallmark target gene sets V1 and V2 are positively enriched in pooled PM and PBM cells versus controls (Fig. 3C,D). Interestingly, the V1 and V2 sets are also positively enriched versus parental FNE1 and P cells in both the PB2 and PB3 lineages, with and without introduction of ectopic MYC (see below). Therefore, while PB3 lineage cells have likely enriched V1 and V2 sets via direct overexpression of endogenous *MYC*, PB2 lineage cells may have also spontaneously upregulated MYC target gene expression via an alternative mechanism, for example by alteration of downstream MYC signalling, as has been observed previously in HGSOC samples (Jiménez-Sánchez et al., 2020). Thus, these observations confirm successful upregulation of *MYC* activity in FNE1 subclones harbouring mutations in *TP53* and *BRCA1*.

### Ploidy analysis reveals independent WGD events

Having established a panel of 18 FNE1 subclones harbouring genetic features found in HGSOC cells (Table 1; Fig. S2A), we set out to determine whether any displayed evidence of CIN. First, we analysed the P1 lineage by flow cytometry to explore changes in ploidy. The *TP53* mutant P1E, the *TP53*/*BRCA1* double-mutant PB1E, plus their MYC-overexpressing counterparts, P1M and PB1M, displayed typical 2c and 4c peaks, indicating no overt deviation from normal ploidy (Fig. S4). By contrast, the *TP53*/*BRCA1* double mutants, PB2E and PB3E, and their MYC-overexpressing counterparts, PB2M and PB3M, displayed 8c peaks, indicating a cycling tetraploid population. In PB2E and PB2M, the 8c peak was small and accompanied by 2c and 4c peaks, suggesting that only a subfraction of the population was tetraploid. In PB3E and PB3M, the 4c and 8c peaks were more apparent than in PB2E/M and an obvious 2c peak was absent, suggesting that the entire population was tetraploid, i.e. had undergone WGD.

Because P1E and P1M appeared overtly normal, mutation of *TP53* alone or in combination with *MYC* overexpression is not sufficient to induce tetraploidization. Moreover, the presence of tetraploidy in PB2E and PB3E also suggests that it arose prior to *MYC* overexpression. Rather, the flow cytometry suggests that the *BRCA1* mutation is possibly driving tetraploidy. And yet, PB1E and PB1M, which also harbour *BRCA1* mutations, do not show evidence of tetraploidy. Note, however, that, as described above, we observed alternative splicing of exon 11 in PB1, and that the BRCA1 deficiency in this line is not as penetrant as in PB2 and PB3 lineages. Nevertheless, the presence of tetraploidy in the PB2 and PB3 lineages suggests independent WGD events in *TP53*/*BRCA1* double-mutant FNE1 cells.

### miFISH confirms WGD and reveals CIN

**Fig. 4.**
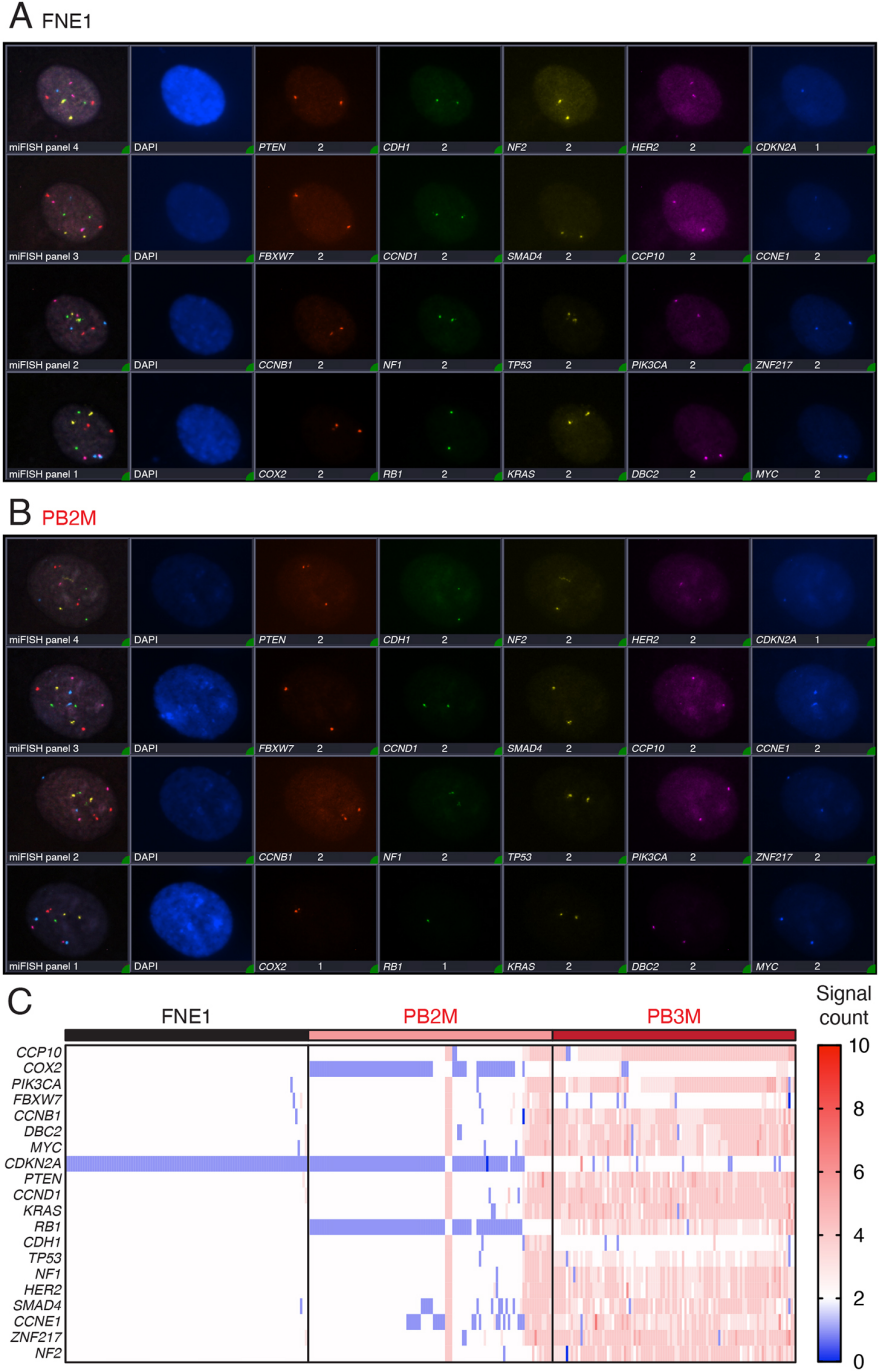
**miFISH implicates ongoing chromosomal instability, aneuploidy and whole-genome doubling in two triple-mutant subclones.** (A,B) Representative composite multiplex, interphase fluorescence *in situ* hybridization (miFISH) images of all 20 probes hybridized in succession on parental FNE1 and PB2M cells, respectively. Note the reduced signal count of *COX2* and *RB1* in PB2M (B) versus parental FNE1 (A). (C) Copy number aberrations of centromere 10 (CCP10) and 19 indicated gene loci in parental FNE1 and the two aneuploid triple-mutant subclones assessed by miFISH. Blue and red indicate copy number loss and gain, respectively, relative to the diploid, parental FNE1. Columns indicate single cells (*n*=100, each for parental FNE1, PB1M and PB3M). P, *TP53* mutant; B, *BRCA1* mutant; M, *MYC*-overexpressing lentivirus. See Fig. S4.

To obtain a more detailed picture of the ploidy changes observed by flow cytometry, we analysed 20 genetic loci in 100 FNE1, PB2M and PB3M cells using multiplex, interphase fluorescence *in situ* hybridization (miFISH) (Heselmeyer-Haddad et al., 2012). In parental FNE1 cells, 19 of the 20 loci analysed were predominantly present in two copies (Fig. 4A,C), consistent with a stable diploid genome, and in line with the scWGS and SKY analyses (Fig. S1). In seven cells, we observed minor abnormalities, with one or two loci deviating from the mode; this, however, is within the margin of error of miFISH performed on cultured cells (Wangsa et al., 2018). By contrast, in every cell analysed, only a single *CDKN2A* signal was detected, indicating a clonal loss of a region on chromosome 9, consistent with the karyotyping (Fig. S1). Note that the *CDKN2A* locus, which encodes the tumour suppressors p16 and p14ARF, is frequently altered in established cell lines and may contribute to their unlimited proliferative potential *in vitro* (Huschtscha and Reddel, 1999).

In contrast to parental FNE1 cells, PB2M and PB3M displayed numerous deviations. As the ploidy measurements by flow cytometry suggested, PB2M harboured both 2c and 4c cells. The 2c subpopulation had the same clonal loss of *CDKN2A*, with additional clonal losses of *COX2* (also known as *PTGS2*) and *RB1* (Fig. 4B,C). These three clonal losses were also present in the 4c subpopulation, with only two foci of each detected. As expected, PB3M was confirmed by miFISH to be entirely composed of 4c cells (Fig. 4C). Like 4c PB2M cells, PB3M cells also had only two signals for some loci, i.e. *COX2*, *FBXW7*, *CDKN2A* and *CDH1*. These losses suggest that either a 4c population of PB3M cells has lost two copies of *COX2*, *FBXW7* and *CDH1*, but not *CDKN2A* (because its baseline is monosomic) or an elusive 2c PB3M population has undergone WGD; we favour the latter explanation. Interestingly, PB3M cells show a pattern of dosage decrease of chromosome 17. In most cells, three copies of *TP53* were detected and four copies of *NF1* and *HER2*. In a subset in which only two *TP53* signals were observed, three copies of *NF1* and *HER2* are seen. Overall, a more diverse pattern of gains and losses was detected in PB2/3M than in FNE1 cells. Thus, these observations confirm independent WGD events in lineages PB2 and PB3. Moreover, the subclonal gains and losses in both diploid and tetraploid backgrounds indicate the acquisition of CIN.

### scWGS reveals CIN in both diploid and tetraploid backgrounds

**Fig. 5.**
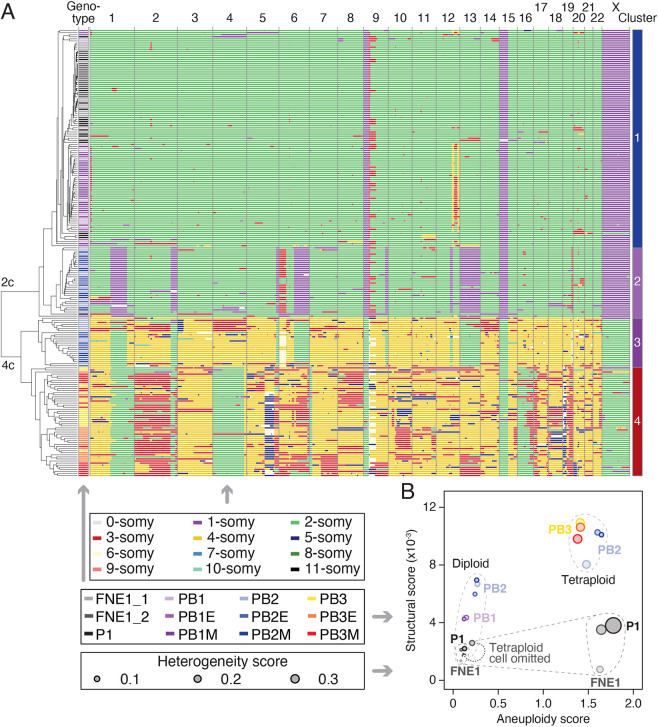
**Single-cell shallow-depth whole-genome sequencing finds ongoing CIN and whole-genome doubling in mutant subclones.** (A) Single cells from indicated genetic backgrounds were subjected to single-cell whole-genome sequencing (scWGS) and subsequent unsupervised hierarchical clustering, which first clusters cells by ploidy and then in a genotype-dependent manner. Autosomes from 1–22 and the X chromosome are displayed as columns. Each row represents a single cell of indicated genetic background (middle box). The colour in each row at a defined genomic location indicates copy number (top box). Note that FNE1_2 is a reproduction of data from Fig. S1C and FNE1_1 and P1 are reproduced in Fig. S5A. (B) Aneuploidy, structural and heterogeneity scores were calculated from scWGS data in A. Structural score is defined as the number of copy number state transitions (within a single chromosome) per Mb, normalized to the number of cells analysed. Generation of heterogeneity and aneuploidy scores was described previously (Bakker et al., 2016)*.* Based on structural and aneuploidy scores, samples separate into a diploid and tetraploid cluster. Note that one of the parental FNE1 samples contained a tetraploid cell (FNE1_1), which resulted in an increase in all three scores, which was reduced if the scores were recalculated omitting that cell (dotted line arrow). Inset in bottom-right corner allows closer inspection of separation of P1 from FNE1 cells. P, *TP53* mutant; B, *BRCA1* mutant; E, empty-vector lentivirus; M, *MYC*-overexpressing lentivirus. See Fig. S5.

Subclonal gains and losses revealed by miFISH indicate CIN in the PB2M and PB3M lines. To explore this in more detail across a wider range of lines, and in particular in an unbiased, genome-wide manner, we performed scWGS-based karyotyping. In addition to parental FNE1 cells, we analysed the *TP53* mutant P1, the three *BRCA1*-deficient derivatives, PB1–3, their *MYC*-expressing subclones, PB1–3M, and the corresponding empty-vector controls, PB1–3E (Fig. S2A). Unsupervised hierarchical clustering identified four karyotype clusters (Fig. 5A). Cluster 1, which exhibited the monosomies at 9p, 15, and X described above (Fig. S1), consisted of parental FNE1 cells, the *TP53* mutant P1 and *TP53/BRCA1* double mutants PB1/E/M. Closer inspection revealed a number of partial or whole chromosome aneuploidies in P1 and PB1/E/M cells. Whereas only two of 35 parental FNE1 cells (5.7%) displayed deviations, ten of 18 P1 cells did so (55.6%), indicating that low-level CIN is already present in *TP53*-deficient P1 cells (Fig. 5A; Fig. S5). Similarly, PB1/E/M cells display low levels of CIN and a clonal, segmental aneuploidy on chromosome 12.

Cluster 2 is characterized by near-diploid genomes with clonal segmental copy number losses on chromosomes 1, 2, 6, 12 and 13, a segmental gain on chromosome 6, and a variety of subclonal gains and losses. By contrast, cluster 3 was dominated by tetrasomies but with segmental disomies on chromosomes 1, 2, 6, 12 and 13, and various subclonal deviations. All the cells in clusters 2 and 3 were from the *TP53*/*BRCA1* double-mutant PB2/E/M cells, thus reflecting the diploid and tetraploid populations identified by miFISH analysis of PB2M. These data also corroborate the *COX2* (1q) and *RB1* (13q) losses seen in PB2M by miFISH, because the corresponding chromosome arms are monosomic in the diploid population. Importantly, because the monosomies in the diploid subpopulation are reflected as disomies in the tetraploid subpopulation, these losses likely occurred prior to the WGD event. The increasing frequency of subclonal deviations in the diploid and tetraploid PB2-lineage populations (68.8% and 78.3% displaying deviations, respectively), compared with P1, indicates exacerbation of the low-level CIN induced by *TP53* loss.

Cluster 4, which is also dominated by tetrasomies, is made up exclusively of cells from the PB3/E/M lineage, reflecting the tetraploid population identified by miFISH analysis of PB3M. Chromosomes 1q, 4 and 16 are disomic, suggesting clonal loss prior to WGD, while many other chromosomes display subclonal whole or segmental gains and losses, indicating pervasive CIN. Indeed, chromosome 5q displays features of rearrangement, loss and amplification. One particular segment is detectable as tetra-, penta- and hexasomy, while the most telomeric region is present as di-, tri- and tetrasomy. Similarly, for chromosome 19, 19p is predominantly detected in five or six copies and 19q is detected most frequently in three copies. Therefore, heterogeneity in the PB3 lineage also indicates that loss of BRCA1 function exacerbated low-level CIN induced by *TP53* loss.

### CIN is initiated by *TP53* loss and exacerbated by *BRCA1* mutation

Taking together, the ploidy analysis, miFISH and scWGS data support a model whereby, in the P1, PB1, PB2 and PB3 lineages, *TP53* mutation initiated low-level CIN on an otherwise diploid background, which was then exacerbated by *BRCA1* mutation, followed by genome-doubling events leading to tetraploidy and more pervasive CIN in PB2/E/M and PB3/E/M cells. Elevated CIN subsequent to tetraploidy was indeed expected based on previous observations in murine mammary epithelial cells (Fujiwara et al., 2005). Whereas both diploid and tetraploid subclones are present in the PB2 lineage, the PB3 lineage is exclusively tetraploid, possibly reflecting an early WGD event during the genesis of this line. Importantly, the extensive CIN generated in our model system is reflective of M-FISH and scWGS from patient-derived *ex vivo* HGSOC cultures, which display profound intercellular heterogeneity with karyotypes characterized by whole-chromosome aneuploidies, rearranged chromosomes, monosomies and tetrasomies (Nelson et al., 2020).

Interestingly, overexpression of *MYC* in the PB1, PB2 and PB3 lineages did not noticeably further exacerbate CIN. Note, however, that PB2/E and PB3/E cells may have spontaneously increased expression of MYC target genes prior to transduction with the *MYC* lentivirus (see below). Thus, it is possible that overexpression of *MYC* targets is contributing to the CIN phenotype in the PB2 and PB3 lineages, but not in the PB1 lineage. These findings are at odds with observations made in RPE-1 cells ectopically expressing MYC and subsequently showing hallmarks of CIN (Rohrberg et al., 2020). However, these observations are based on p53-proficient cells and cell-biological readouts, not scWGS. Thus, to disentangle the relationship between *BRCA1* loss and *MYC* overexpression in more detail, cell-biological assays in PB/E and PBM cells, as well as scWGS, of P1–3M are required.

### *TP53* loss initiates extensive transcriptional rewiring

The observation that *TP53* mutant cells accumulate aneuploidies was surprising considering the longstanding observation that p53-null HCT116 cells remain diploid (Bunz et al., 2002; Thompson and Compton, 2010). Indeed, we also found that CRISPR-generated *TP53^−/−^* HCT116 cells do not develop aneuploidies (Simões-Sousa et al., 2018). While *TP53* loss in HCT116 and RPE-1 cells can facilitate tolerance of abnormal karyotypes, p53 activation in response to aneuploidy is not consistent and is context dependent (Santaguida et al., 2017; Simões-Sousa et al., 2018; Soto et al., 2017; Thompson and Compton, 2010). Moreover, note that such aneuploidy tolerance studies utilized experimental induction of chromosome mis-segregation in cells lacking p53. However, the emergence of aneuploid clones with *TP53* loss has been observed in untreated mammary epithelial and RPE-1 cells (Kok et al., 2020; Salehi et al., 2021; Soto et al., 2017). In addition, multiple cellular processes were deregulated in response to p53 inactivation in transformed murine embryonic fibroblasts, including ploidy control (Valente et al., 2020). Therefore, the fact that *TP53* mutant FNE1 cells accumulate aneuploidies without exposure to exogenous replication stress or mitotic perturbation suggests that, in this context, p53 loss is also sufficient to initiate CIN. To explore potential underlying mechanisms, we performed global transcriptomics, analysing the panel of 18 derivatives by RNAseq. Parental FNE1, P1, P1E and P1M were analysed in triplicate, totalling 27 samples.

**Fig. 6.**
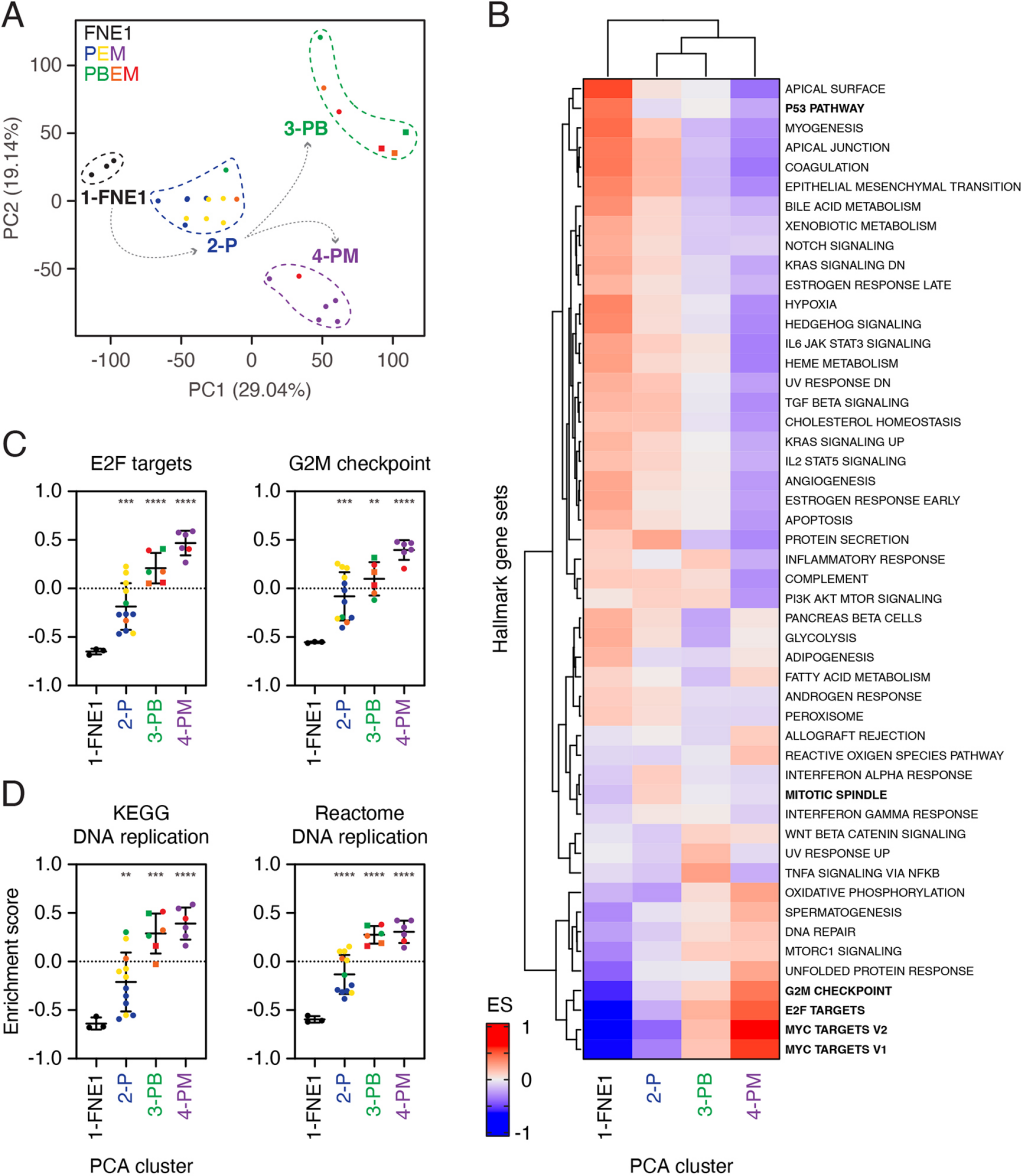
**Transcriptome profiling reveals cell cycle deregulation upon p53 loss.** (A) Principal component analysis (PCA) of 27 cell lines analysed by RNAseq separates parental FNE1 samples from mutant subclones and BRCA1-deficient subclones from those with fully or partially functioning BRCA1. Indicated colours correspond to sample genotype. Dashed lines capture four clusters defined by similarity of transcriptomes that broadly follow sample genotype, except for PB1 and PB1E/M (see text). Samples derived from the PB3 lineage are depicted as squares. Percentage variance of principal components 1 (PC1) and 2 (PC2) are indicated in parentheses along axes. See Table S3 for input data. (B) Gene set variation analysis (GSVA) was performed on samples grouped according to each of the four distinct PCA clusters and the mean used to perform unsupervised hierarchical clustering. The 50 Hallmark gene sets are indicated, and the enrichment score (ES) is depicted in blue or red for negative or positive enrichment, respectively. See Fig. S6 and Table S4. (C,D) Results from two representative Hallmark gene sets from B (C), and the DNA replication gene sets from the KEGG (D, left) and Reactome (D, right) collections are shown. Samples were grouped based on PCA cluster allocation and the colour of individual data points corresponds to sample genotype as in A. Samples derived from the PB3 lineage are depicted as squares. For cluster 1 (FNE1), *n*=3 samples; cluster 2 (P), *n*=12; and clusters 3 and 4 (PB and PM), *n*=6. Horizontal bar and error bars indicate mean and s.d., respectively. Asterisks depict adjusted *P*-values between indicated groups compared with cluster 1 (FNE1) by Brown–Forsythe and Welsh ANOVA, where **adjusted *P*≤0.005, ***adjusted *P*≤0.0005 and ****adjusted *P*<0.0001. P, *TP53* mutant; B, *BRCA1* mutant; E, empty-vector lentivirus; M, *MYC*-overexpressing lentivirus. See Fig. S7 and Table S5.

Principal component analysis (PCA) yielded four clusters, with cluster 1 consisting of the three parental FNE1 samples (Fig. 6A). Cluster 2 is dominated by the three independent *TP53* mutants, P1–3, and their empty-vector derivatives P1–3E, thus reflecting gene expression changes induced by *TP53* loss. Cluster 3 contained the PB2 and PB3 lineages, reflecting the effect of *BRCA1* loss in the *TP53*-mutant background. Cluster 4 contained P1–3M and thus reflects gene expression changes induced by *MYC* overexpression on the *TP53*-mutant background. Note that PB1, and its empty-vector derivative PB1E, are in cluster 2, rather than the *BRCA1*-deficient cluster 3. Likewise, PB1M is in cluster 4 with P1–3M. This is consistent with PB1/E/M cells falling into cluster 1 of the scWGS analysis with P1 and FNE1 cells. Note also that although *MYC* overexpression had a marked effect on P1–3 and PB1, it had little effect on PB2 and PB3. However, again, as described above, these cells appear to have spontaneously upregulated *MYC-*target expression (see below), explaining why ectopic *MYC* had little additional effect. Based on these observations, we conclude that *TP53* mutation alone results in profound transcriptional rewiring, which is further amplified by either elevated MYC activity or *BRCA1* loss; in the latter case, spontaneous MYC upregulation and MYC-independent enrichment of target genes were observed.

### *TP53* loss deregulates cell cycle gene expression programmes

To determine how *TP53* and *BRCA1* loss and *MYC* overexpression deregulate the transcriptome in FNE1 cells, we performed gene set variation analysis (GSVA) using the Hallmark gene set collection, an approach that allows comparisons across multiple sample groups (Hänzelmann et al., 2013). Unsupervised hierarchical clustering of the 27 samples resulted in a similar separation as the PCA, with parental FNE1 (cluster 1) and the *TP53* mutants (cluster 2) forming one clade (Fig. S6). The *TP53* mutants overexpressing *MYC* (cluster 4) formed a separate clade, while the *BRCA1*-deficient lineages PB2 and PB3 (cluster 3) formed a further two clades. Next, we grouped the various cell lines into the four PCA clusters and interrogated specific gene sets. Note, that we included PB1/E cells in the P cluster and PB1M cells in the PM cluster based on their associations with these groups by PCA, hierarchical clustering and scWGS. Consistent with p53 proficiency, the p53 pathway gene set was positively enriched in the parental FNE1 group (cluster 1) versus the *TP53*-mutant lineages (clusters 2–4; Fig. 6B; Fig. S7). *MYC* target gene sets V1 and V2 were most highly positively enriched in cluster 4, i.e. the *TP53*-mutant samples overexpressing *MYC* (Fig. 6B; Fig. S7). *MYC* targets were also enriched in the PB2 and PB3 lineages (cluster 3), despite only two of the six lines harbouring ectopic *MYC*, demonstrating spontaneous upregulation of *MYC* targets in PB2 and PB3. E2F targets, G2/M checkpoint and mitotic spindle gene sets also stand out; in all three cases, parental FNE1 cells (cluster 1) display negative enrichment, which suggests attenuation of these genes' expression in a p53-proficient background. Consequently, as genetic manipulations are introduced, the enrichment score progressively increases (clusters 2–4; Fig. 6C; Fig. S7). Importantly, because cluster 2 cells showed significant increases in enrichment score versus parental FNE1 cells for E2F targets, MYC targets, G2/M checkpoint and mitotic spindle gene sets, these observations indicate that loss of p53 is sufficient to deregulate multiple aspects of cell cycle control (Fig. 6C; Fig. S7). Conversely, this reveals a surprising role for wild-type p53; in the absence of cellular stresses predicted to hyperstabilize p53, basal levels of p53 appear to be, either directly or indirectly, repressing expression of genes governing a range of cell cycle controls.

### *TP53* loss deregulates expression profiles of DNA replication genes

As replication stress is an established CIN driver (Burrell et al., 2013; Tamura et al., 2020), we next asked whether evidence of replication stress manifested in the RNAseq data. Indeed, upregulation of DNA replication genes is an established mechanism to tolerate replication stress (Bianco et al., 2019). However, because the Hallmark collection does not contain a DNA replication gene set, we analysed the DNA replication gene sets from the Kyoto Encyclopedia of Genes and Genomes (KEGG) and Reactome collections. GSVA revealed that the DNA replication gene sets showed significant increases in enrichment score versus parental FNE1 cells (Fig. 6D). Although the enrichment score remains negative for the *TP53* mutants (cluster 2), it is significantly increased compared with that of parental FNE1 cells, indicating that p53 loss is perhaps sufficient to induce replication stress. Indeed, the enrichment score of DNA replication gene sets increased in a similar manner to the other gene sets above, and is greatest in PM samples, consistent with MYC overexpression precipitating DNA replication stress (Kotsantis et al., 2018).

Taken together, our observations indicate that *TP53* mutation is sufficient to deregulate multiple cell cycle gene expression programmes and trigger transcriptional alterations consistent with a response to replication stress, and that these changes are exacerbated by *BRCA1* mutation and *MYC* overexpression. Coupled with the ploidy and karyotype analyses, these observations provide a plausible mechanism by which *TP53* loss is sufficient to initiate CIN in FNE1 cells.

### p53-deficient mouse fallopian tube organoids display cell cycle deregulation

Our finding that *TP53* loss is sufficient to deregulate gene expression programmes governing cell cycle progression, DNA replication and mitosis was surprising. Therefore, we asked whether data from an independent model system supported our observation. Recently, a series of mouse fallopian tube organoids has been developed harbouring conditional alleles designed to inactivate *Trp53* and express an SV40 large T antigen, which in turn suppresses Rb1 function (Zhang et al., 2019). Utilizing the publicly available RNAseq data, we analysed differentially expressed genes and performed GSEA analysis. PCA shows that the wild-type and mutant organoids form two distinct clusters, indicating divergent gene expression profiles (Fig. S8A, Tables S6 and S7), and unsupervised hierarchical clustering analysing E2F, G2/M and mitotic spindle-related genes clearly separated wild-type from mutant organoids (Fig. S8B). Finally, we correlated the normalized enrichment scores for various gene sets in our human FNE1-derived *TP53*-deficient P cells with the mouse organoid samples. This showed that MYC targets, E2F targets, G2/M checkpoint genes and mitotic spindle genes were all positively correlated in both samples (Fig. S8C). Thus, although the mouse organoids are deficient for both p53 and Rb1 function, the gene expression changes are mirrored in human FNE1 cells harbouring mutant *TP53*, further supporting our notion that p53 loss in human FNE1 cells is sufficient to drive profound transcriptional deregulation of cell cycle regulators.

### *TP53* loss confers tolerance to pharmacologically induced mitotic perturbation

Our observations show that, in FNE1 cells, *TP53* mutation is sufficient to induce CIN, and that this is accompanied by deregulation of gene expression networks required to maintain chromosomal stability. As gene expression profiling only indirectly reflects cell function, we asked whether *TP53* mutation does indeed modulate the functionality of chromosome stability pathways. To do this, we challenged parental FNE1 cells and *TP53*-deficient P1 cells with GSK923295, an inhibitor of the mitotic kinesin CENP-E (CENP-Ei), and analysed the effects by time-lapse microscopy, using cell confluency as a proxy for proliferation. Note that pharmacological inhibition of CENP-E prevents congression of a small number of chromosomes, thus preventing satisfaction of the spindle assembly checkpoint (SAC), in turn inducing a mitotic arrest. Eventually, ‘SAC exhaustion’ results in anaphase onset and mitotic exit in the presence of polar chromosomes, leading to aneuploidy (Bennett et al., 2015; Wood et al., 2010).

**Fig. 7.**
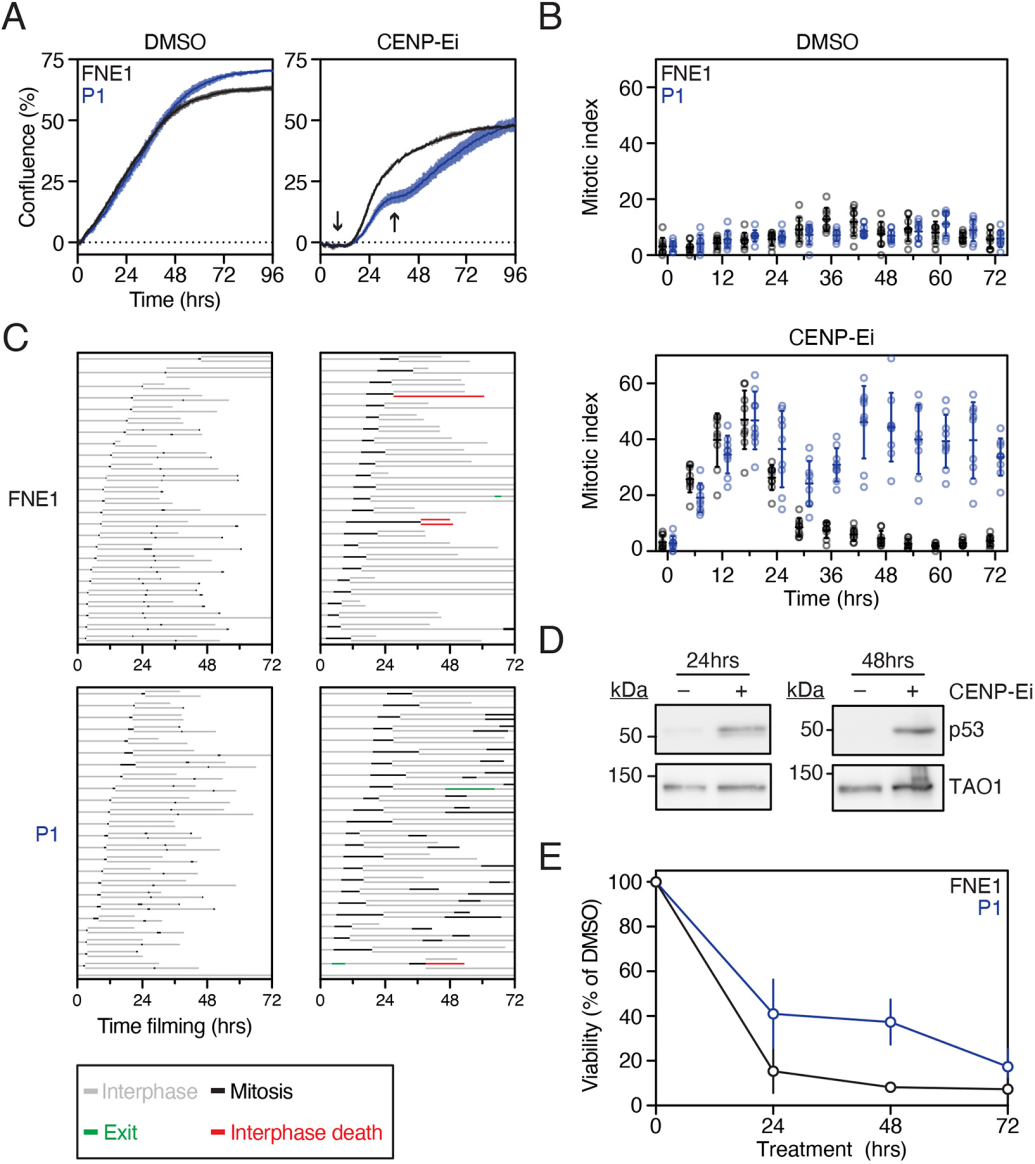
**p53 loss alone permits pharmacologically induced CIN.** (A) Confluence curves of parental FNE1 and *TP53*-mutant (P1) cells in the presence of DMSO (vehicle) or CENP-Ei (GSK923295). Confluence was normalized to *t*_0_ by subtraction. Arrows indicate mitotic arrest. Representative results from three technical replicates of at least three independent experiments. Error bars represent s.d. (B) Mitotic index was profiled in parental FNE1 and P1 cells in the presence of DMSO or CENP-Ei at indicated time points. Results shown are from three fields of view from three technical replicates shown in A. (C) Cell-fate profiling by time-lapse microscopy of parental FNE1 and P1 cells in the presence of DMSO or CENP-Ei. 25 cells and both daughters of the first mitosis were profiled per condition. (D) Immunoblot of p53 expression in parental FNE1 cells treated with DMSO or CENP-Ei for 24 h and 48 h. TAO1 serves as loading control. (E) Crystal Violet-based viability assay of parental FNE1 and P1 cells treated with DMSO or CENP-Ei for indicated time periods followed by drug washout. Experiment was concluded 14 days after drug addition, and viability was normalized to DMSO-treated cells. Two independent experiments are shown for the 24- and 72-h washouts and three for 0- and 48-h washouts. Error bars represent s.d.

In the absence of inhibitor, both populations proliferated and then reached a confluency plateau after 48 h (Fig. 7A). Upon exposure to CENP-Ei, both parental FNE1 and P1 cells underwent mitotic arrest, evidenced by a static confluence during the first 12 h and an increase in mitotic index (Fig. 7A,B). They eventually divided and flattened out, resulting in a confluence increase. Parental FNE1 cells failed to divide again, yielding a long second plateau and progressive decrease in mitotic index. By contrast, *TP53*-mutant P1 cells entered and exited a second mitosis, indicated by a short second plateau followed by sustained confluency increase and consistently increased mitotic index (Fig. 7A,B). To confirm this, we performed cell-fate profiling, analysing 25 individual cell divisions and tracking the fate of the daughters. In the absence of CENP-Ei, cells in both populations completed multiple rounds of cell division (Fig. 7C). Upon exposure to CENP-Ei, both parental FNE1 and P1 cells underwent prolonged mitotic delays (Fig. 7C, compare the lengths of the black bars), but, following eventual exit, while parental FNE1 cells were then blocked in the subsequent interphase, the vast majority of p53-deficient P1 cells entered second mitoses, indicating continued cell cycle progression.

Consistent with the interphase block, p53 was stabilized in parental FNE1 cells (Fig. 7D) and longer-term viability was diminished (Fig. 7E). Thus, we conclude that loss of *TP53* in FNE1 cells is sufficient to compromise the postmitotic cell cycle blocks that would normally prevent proliferation of aneuploid daughter cells following a prolonged mitosis and chromosome mis-segregation event. Although we have not analysed the effect of p53 loss on replication stress and G2/M checkpoint controls directly, these observations are consistent with the notion that *TP53* disruption is sufficient to compromise cell biological processes that would otherwise function to minimize CIN.

## DISCUSSION

HGSOC is characterized by ubiquitous mutations in *TP53* and high levels of aneuploidy as a consequence of CIN (Cancer Genome Atlas Research Network, 2011; Ciriello et al., 2013). However, a genetic basis for CIN in HGSOC remains elusive. In this study, we set out to investigate whether genetic alterations commonly observed in HGSOC are sufficient to drive CIN, in the HRD group characterized by *BRCA1/2* mutation and *MYC* amplification (Wang et al., 2017). As HGSOC predominately originates from the fallopian tube, we generated a panel of CRISPR/Cas9-mutant, fallopian tube-derived subclones based on the *hTERT*-immortalized, non-transformed cell line FNE1 (Labidi-Galy et al., 2017; Merritt et al., 2013). We first showed that FNE1 cells mount a robust p53 response indicating pathway proficiency, in contrast to other model cell lines that rely on p53 suppression for immortalization (Fig. S1A,B) (Karst and Drapkin, 2012; Karst et al., 2011; Nakamura et al., 2018). Importantly, parental FNE1 p53 proficiency allowed us to directly test the impact of p53 loss of function alone, and in combination with *BRCA1* deficiency and *MYC* overexpression, in an isogenic model system. Using this system, we find that p53 loss alone is sufficient to cause aneuploidy in FNE1 cells, which is exacerbated in the absence of functional BRCA1. Analysing the transcriptome revealed that cell cycle deregulation was apparent in *TP53* single mutants and amplified in *TP53*/*MYC* double mutants. The most highly enriched gene sets compared with parental FNE1 cells were G2/M checkpoint, E2F targets, DNA replication and mitotic spindle, which were enriched in cells deficient for p53 alone and progressively more enriched with additional genetic manipulations. These findings, which were consistent with publicly available data from mutant mouse fallopian tube organoids (Fig. S8) (Zhang et al., 2019), therefore indicate that p53 loss alone results in transcriptional changes that can deregulate the cell cycle and promote low-level CIN. Because truncating mutations that lead to a p53 loss of function only account for 35% of HGSOC (Cancer Genome Atlas Research Network, 2011), future work will require investigating mis-sense and potential gain-of-function *TP53* mutations in this context. Additionally, modelling these genetic changes in other human systems such as alternative non-transformed immortalized cell lines or organoids is required to corroborate our observations.

*TP53* mutations have been firmly established as early and ubiquitous events in HGSOC development. However, the implications of *TP53* mutation on fallopian tube epithelial cells remain poorly understood and have thus been highlighted as key to understanding HGSOC development (Bowtell et al., 2015). Although p53 is an established suppressor of proliferation in response to aneuploidy, mutations in *TP53* correlate consistently and most strongly with aneuploidy and WGD in multiple tumour types (Bielski et al., 2018; Ciriello et al., 2013; Davoli et al., 2017; Taylor et al., 2018; Thompson and Compton, 2010; Zack et al., 2013). Although evaluation of fallopian tube-derived models with suppressed p53 previously suggested that additional p53-independent mechanisms act as barriers to proliferation of aneuploid cells, the same study found increased transformation potential with p53 suppression in combination with pharmacologically induced aneuploidy in soft agar assays (Chui et al., 2019). Conflicting observations have also been reported regarding the relationship between p53 loss and the emergence of aneuploidy in colorectal cancer cell lines (Bunz et al., 2002; Simões-Sousa et al., 2018). Indeed, we observed an increase in structural and numerical aneuploidy by scWGS when comparing parental FNE1 with p53-deficient P1 cells. Although the magnitude of this change is moderate quantitatively, on a qualitative level it is evident that P1 cells harbour more whole-chromosome or chromosome arm aneuploidies than parental FNE1 cells from two different passages (Fig. 5; Fig. S5). Therefore, mounting evidence from us and others suggests that p53 loss alone may be sufficient to induce low-level CIN, permitting cells to develop karyotypic heterogeneity. However, the importance of environmental factors such as oxygen levels has only recently been brought to light, which might impact chromosome segregation and the processes preceding mitosis, as well as the selection of viable karyotypes. It is conceivable that growth at atmospheric oxygen may previously have masked the emergence of aneuploidy, as euploid cells would outcompete aneuploid cells more rapidly than under normoxic or hypoxic conditions (Rutledge et al., 2016).

The development of isogenic mutant cell lines allowed us to study mitotic perturbations side by side in p53-proficient and -deficient cells. HGSOC is appreciated as one of the most chromosomally unstable cancers based on *in silico* analyses of cancer genomes backed up by cell biological studies of mitosis in HGSOC models (Nelson et al., 2020; Tamura et al., 2020). Primary cultures established from HGSOC patients' ascitic fluid can take more than 6 h to complete mitosis in extreme cases, and up to 24 h in select examples of individual cells (Nelson et al., 2020). This dramatically increased mitotic duration compared with non-transformed cells has been shown to be limited in a p53-dependent manner termed the ‘mitotic timer’. Indeed, knockout of *TP53* and its upstream regulators in this specific context, *USP28* and *53BP1* (also known as *TP53BP1*), rescued growth arrest following prolonged mitosis of up to 6 h (Lambrus et al., 2016). Inhibiting the mitotic kinesin CENP-E pharmacologically, we could achieve a comparable increase in mitotic duration and were able to show that p53 was stabilized in response to CENP-Ei. Furthermore, we show that P1 cells tolerate this stress better than parental FNE1 cells, in short-term as well as long-term assays (Fig. 7). Thus, we show that p53 loss precipitates low-level CIN and also partially rescues viability upon mitotic delay and chromosome mis-segregation; this dual- or potentially multi-functionality of p53 provides an explanation as to why one of the most chromosomally unstable tumour entities is characterized by ubiquitous *TP53* mutations. From a technical perspective, these experiments set the scene for further analyses of mitosis in response to pharmacological perturbations, such as routinely used chemotherapeutics, using our panel of genetically defined fallopian tube epithelial cells.

Beyond mutations in *TP53*, mutations in *BRCA1*/*2* are the second most common mutation in HGSOC (12% of cases each). In genetically engineered mouse models of mammary epithelial cancer, deletion of exon 11 of *BRCA1* was shown to cause functional G2/M checkpoint disruption and tumorigenesis (Weaver et al., 2002; Xu et al., 1999). Based on these two observations, and because human BRCA1-deficient fallopian tube-derived cell line models are lacking, we mutated *BRCA1* to create a model of more pronounced CIN and HRD. We found that our three cell lines deficient in full-length BRCA1 are distinct from one another; based on the analysis of gene expression profiles by PCA and GSVA, PB1 clusters with P cells and PB2 and PB3 form an independent cluster. This distinction likely reflects biological heterogeneity following *BRCA1* mutagenesis that led to exacerbation of CIN. Indeed, PB1 cells are largely 2c, while PB2 cells harbour a 2c and 4c population, and PB3 cells are 4c. Interrogation of RNAseq at the nucleotide level found that PB2 and PB3 have an identical exon 3 mutation; however, PB1 cells express a splice variant of exon 11 as a consequence of a mutation in the same exon, which is known to diminish PARPi sensitivity versus other BRCA1 mutants over time (Wang et al., 2016). Our findings agree with this *BRCA1* variant having some functionality, in that, despite the absence of full-length *BRCA1*, its retained expression is sufficient to protect against aneuploidy. As flow cytometric and miFISH evidence suggested aneuploidy, PB2 and PB3 cells were subjected to scWGS and indeed the extent of copy number heterogeneity observed exceeded that of P1 and PB1E/M cells. Interestingly, we observed a propensity for WGD, which is associated with poor prognosis, in both PB2 and PB3 cells, despite *BRCA1* mutations being associated with good prognosis and not being reported to correlate with WGD (Bielski et al., 2018). Although this could reflect an *in vitro* selection pressure permitting the detection of 4c PB2 and PB3 cells in our system, similar observations were recently reported in *TP53/BRCA1* double-mutant mammary epithelial cells (Funnell et al., 2021 preprint). Nevertheless, we conclude that the combination of p53 and BRCA1 deficiency can drive CIN in a context-dependent manner, where low levels of BRCA1 activity such as observed in PB1 remain protective. Dissecting the order of events in a time-dependent manner could lend additional insights into CIN and WGD in HGSOC. As WGD is associated with drug resistance, separating 2c from 4c PB2 cells could also aid in dissecting the role of WGD in PARPi sensitivity (Kuznetsova et al., 2015).

Several non-genetic causes of CIN such as increased microtubule assembly rates, centrosome amplification and replication stress have been identified in colorectal cancer and HGSOC cell lines (Bastians, 2015; Tamura et al., 2020). To decipher the causes of CIN in our mutant subclones, we turned to analysis of transcriptomics, which enabled us to take an unbiased, genome-wide approach. We observed that loss of p53 alone resulted in an enrichment of gene sets composed of genes regulating the cell cycle and DNA replication, consistent with previous observations in HCT116 cells (Allen et al., 2014). We suggest that this effect is a consequence of the downregulation of canonical p53 targets such as *MDM2* and *CDKN1A*, which encodes the CDK inhibitor p21 (Fig. 4C). p21 plays an important role in suppressing S-phase entry by negatively regulating cyclin E and CDK2. The absence of this negative regulation thus permits cyclin E and CDK2 to hyperphosphorylate RB1 more rapidly, which results in de-sequestration of E2F, a key transcription factor controlling S-phase entry (Sullivan et al., 2018). Indeed, the E2F target gene set is significantly less negatively enriched in P samples than in parental FNE1 samples (Fig. 6C). To contextualize, p21 has been shown to protect cells from CIN. In a genetically engineered mouse model of p53 separation of function, which was apoptosis deficient but partially functional to suppress cell cycle progression, deletion of p21 led to an increase in CIN (Barboza et al., 2006). Moreover, three of the four sample groups showed significantly different and more positive enrichment scores in cell cycle-related gene sets compared with parental FNE1 cells.

Except for the mitotic spindle gene set, overexpression of *MYC* consistently amplified the already observed enrichment in p53-deficient P samples, likely reflecting the role of MYC as transcriptional amplifier (Lin et al., 2012; Nie et al., 2020, 2012). This held true also for the negative enrichment of the p53 pathway gene set, where P samples displayed an already negative enrichment score that was even more negative in the PM samples (Fig. S7). In contrast to P samples, *MYC* overexpression did not seem to have the same impact on the transcriptome in PB2 and PB3 as it did in PM samples (Fig. 6A). In fact, PB2/E and PB3/E samples showed more positive enrichment of MYC targets V1 and V2 than P samples even without *MYC* overexpression; this is consistent, at least in PB3 samples, with higher endogenous *MYC* transcript levels, whereas we speculate that PB2/E cells have spontaneously upregulated MYC target genes in the absence of elevated MYC expression (Table 1). Interestingly, the PB2M sample reaches the highest enrichment score of the PB2 lineage, suggesting that ectopic MYC is active in this sample, but perhaps to a lesser extent than in the PM samples. Consistent with our findings, proteogenomic analyses of HGSOC have suggested a causal role for the deregulation of mitotic and DNA replication genes in the extensive CIN observed in this disease; however, the causes of this deregulation could not be definitively dissected in patient samples (McDermott et al., 2020). Taking these data into account, we suggest that CIN is caused by the cumulative changes in cell cycle regulators' expression, rather than a single causative gene, as a consequence of e.g. loss of p53 signalling through its downstream effector p21, which promotes transcriptional programs of cell cycle progression. Future work should focus on genetic add-back experiments of downregulated *CDKN1A* to investigate whether this rescues the observed deregulated expression of cell cycle genes and low-level CIN.

In summary, we provide evidence, based on a novel human, fallopian tube-derived cell line panel that p53 loss leads to transcriptomic deregulation of cell cycle regulators, which is amplified by the loss of the tumour suppressor *BRCA1* and overexpression of the oncogene *MYC*. We propose that the sum of these transcriptional changes causes CIN in HGSOC and show that P1 cells display low levels of aneuploidy. Furthermore, we show that additional genetic manipulation of *BRCA1* exacerbated both the enrichment of cell cycle regulators and aneuploidy. Finally, p53 loss increased tolerance of pharmacological perturbation of mitosis using an inhibitor of CENP-E, further supporting its potential role in the development of CIN in HGSOC. Our data point to the dual- or multi-functional role of p53 whereby its loss precipitates CIN by causing cell cycle and DNA replication deregulation, while simultaneously promoting the survival of aneuploid cells that experienced those stresses in the previous cell cycle.

## MATERIALS AND METHODS

Details of critical commercial reagents and kits, drugs, antibodies, recombinant DNA, oligonucleotides, FISH probes and software are provided in Table S1.

### Cell culture

FNE1 cells (a kind gift from Dr Tan A. Ince, Brooklyn Methodist Hospital, Weill Cornell Medicine, New York, NY, USA) were cultured in WIT-Fo culture medium (FOMI) at 5% O_2_ and 5% CO_2_ at 37°C, as described previously (Merritt et al., 2013). AAV293T cells (American Type Culture Collection) were cultured in Dulbecco's modified Eagle medium supplemented with 10% foetal bovine serum and 100 U/ml penicillin-streptomycin, at atmospheric O_2_ and 5% CO_2_ at 37°C. All cell lines were authenticated using a Promega Powerplex 21 System and regularly tested for mycoplasma either by PCR (both at CRUK Manchester Institute Molecular Biology Core Facility) or Lonza enzymatic test (Animal Molecular Diagnostics Laboratory at the National Cancer Institute (NCI), Frederick, MD, USA).

Lentiviruses were produced by co-transfection of AAV293T cells at 5×10^4^ cells per well in a 24-well microplate with recombinant DNA at 0.375 μg lentivirus of interest, 0.5 μg psPAX2 and 0.125 μg pMD2.G (both kind gifts from Dr Didier Trono, EPFL, Lausanne, Switzerland) using a Promega ProFection Mammalian Transfection System kit according to manufacturer instructions. Transfection medium was replaced after overnight incubation and lentivirus was harvested every other day for 4 days. Supernatant containing lentivirus was centrifuged, filtered (0.45 μm) and frozen for storage at −80°C.

CRISPR/Cas9-expressing FNE1 cells were generated by transduction with Dharmacon Edit-R Inducible Lentiviral Cas9 particles followed by selection with blasticidin S at 8 μg/ml. Cas9 expression was assessed by titrating tetracycline and induced using 15 μg/ml in subsequent experiments. To mutate *TP53*, FNE1 cells expressing inducible Cas9 were transduced with lentiGuide-Puro [a kind gift from Dr Feng Zhang (Sanjana et al., 2014)] containing a guide RNA (gRNA) targeting *TP53* (Table S2) and selected in 0.7 μg/ml puromycin. Cas9 was then induced for 5 days before isolation of single-cell clones by limiting dilution (either immediately or following 5 days further selection in Nutlin-3). Taking P1 cells forward, cells were transduced with six different lentiGuide-Neo (see ‘Molecular Biology’ section for details) lentiviruses each containing a unique gRNA targeting *BRCA1* (Table S2). After neomycin selection at 0.8 mg/ml, Cas9 was induced as above before isolation of single-cell-derived subclones by limiting dilution. Clones were screened by immunoblotting (see ‘Cell biology’ section for details). Mutations in targeted genes were assessed in the RNAseq dataset using Integrative Genomics Viewer (Version 2.8.0) and annotated according to standard practices (Ogino et al., 2007; Robinson et al., 2011). Mutations in *BRCA1* in PB1 and PB2 cells were confirmed using Sanger sequencing. *MYC*-overexpressing and cognate ‘E’ cells were generated by transduction with pLenti CMV Hygro DEST or MYC lentiviruses [a kind gift from Drs Eric Campeau and Paul Kaufman (Campeau et al., 2009)] and selection with 25 μg/ml hygromycin, maintaining a polyclonal cell population. Immunoblotting and RNAseq were employed to confirm functionality of *MYC* overexpression. All lentiviral transductions were performed in 4 μg/ml polybrene.

Functional deficiency of p53 and BRCA1 in putative clones was confirmed by exploiting the known synthetic-viable and -lethal relationships with Nutlin-3 and Olaparib treatment, respectively. Nutlin-3 assays were performed by seeding 30,000 cells (parental FNE1, P1 and P3 transduced with pLVX mCherry-H2B Puro) into Primaria 24-well microplates. The next day, either vehicle [dimethyl sulfoxide (DMSO)] or 10 μM Nutlin-3 (Selleck Chem, Houston, TX, USA) were added in Phenol Red-free medium and the cells imaged for 96 h on an IncuCyte^®^ ZOOM (Satorius AG, Göttingen, Germany) time-lapse microscope housed in a low-oxygen incubator (5% O_2_, 5% CO_2_). IncuCyte^®^ ZOOM custom software was used in real time to measure confluency and red fluorescent object count and for data analysis. Population doubling for each culture was calculated by performing a log_2_ transformation of the fold-change nuclear count from *t*_0_ and plotted against time. PARPi (Olaparib, Selleck Chem) sensitivity was assessed by seeding 100 cells directly into drug- or vehicle-containing medium in collagen-coated, black 96-well microplates (Greiner Bio-One North America, Monroe, NC, USA). Media and drug were replenished every 3 days. On day 7, 30 μl CellTiter-Blue^®^ (Promega, Madison, WI, USA) reagent were added to 150 μl medium and incubated for 4 h followed by fluorescence signal measurement on a SpectraMax M2 plate reader (Molecular Devices, San Jose, CA, USA).

Assays studying the response to CENP-E inhibition were performed using GSK923295 (Selleck Chem). For live-cell imaging, 30,000 cells were seeded into Primaria 24-well microtitre plates, allowed to adhere overnight, vehicle or drug (250 nM) were added the next day, and imaging on an IncuCyte^®^ ZOOM time-lapse microscope was performed, as described above. Cell-fate profiles were analysed manually based on exported MPEG-4 videos. Long-term viability assays were performed by seeding 2000 cells into Primaria six-well microtitre plates, allowing the cells to adhere overnight and adding vehicle or drug the next day. Drug washout was performed at indicated time points and medium replenished every 36–48 h. Experiments were concluded after 14 days, cells were washed, fixed with 1% formaldehyde (in PBS) and stained with Crystal Violet (0.05% in dH_2_O). Quantitation was achieved by extracting Crystal Violet with acetic acid and measuring absorbance on a SpectraMax M2 plate reader.

A summary of all cell lines generated is provided in Table 1 and Fig. S2A.

### Cell biology

Cells were harvested normally or *in situ*, lysed in sample buffer (0.35 M Tris-HCl pH 6.8, 0.1 g/ml sodium dodecyl sulphate, 93 mg/ml dithiothreitol, 30% glycerol, 50 mg/ml Bromophenol Blue) and boiled for 5 min. Proteins were resolved by SDS-PAGE and electroblotted by wet transfer onto Immobilion-P membranes (Millipore Sigma, Burlington, MA, USA). Membranes were blocked in 5% milk in TBS-T (50 mM Tris-HCl pH 7.6, 150 mM NaCl, 0.1% Tween-20) and incubated with primary antibodies at indicated concentrations (Table S1) overnight at 4°C. Membranes were then washed with TBS-T and incubated with horseradish-peroxidase-conjugated secondary antibodies (Table S1) for 2 h at room temperature. After further washes with TBS-T, detection was performed using EZ-ECL Chemiluminescence Substrate (Biological Industries, Kibbutz Beit-Haemek, Israel, part of the Satorius group) or Luminata Forte Western HRP Substrate (Millipore Sigma). Membranes were imaged on BioSpectrum 500 (UVP, Upland, CA, USA) imaging system.

For p53 immunofluorescence, parental FNE1 cells were seeded onto collagen-coated 19 mm coverslips, incubated overnight and treated with 10 μM Nutlin-3 for 8 h. For RAD51 immunofluorescence, FNE1, P1, PB1, PB2 and PB3 cells were seeded as aforementioned and treated with 2 Gy X-rays (CellRad^®^, Faxitron^®^) and 1 μM Olaparib. Twenty-four hours later, the cells were washed with PBS, fixed (1% formaldehyde in PBS), quenched with glycine, permeabilized with PBS-T (PBS, 0.1% Triton X-100), incubated consecutively with primary [mouse anti-p53, DO-1, Santa Cruz Biotechnology, Dallas, TX, USA; rabbit anti-RAD51, BioAcademia, Osaka, Japan; sheep anti-CENP-F, Hussein and Taylor (2002)] and secondary (donkey anti-mouse conjugated with Cy3; donkey anti-rabbit conjugated with Cy2; donkey anti-sheep conjugated with Cy3; all polyclonal from Jackson ImmunoResearch, West Grove, PA, USA) antibodies for 30 min each with a wash step in between (Table S1). Coverslips were then washed with PBS-T, stained with Hoechst 33258 (Millipore Sigma), washed with PBS-T and mounted onto slides (90% glycerol, 20 mM Tris-HCl pH 9.2). Slides were imaged on an Axioskop2 microscope fitted with a 40× objective (both from Zeiss, Jena, Germany) and a CoolSNAP HQ camera (Teledyne Photometrics, Tucson, AZ, USA) operated by MetaMorph software (Molecular Devices). Image analysis was performed with Adobe Photoshop^®^ CC 2015 (Adobe Systems, Los Angeles, CA, USA). For RAD51 foci formation assays, cells staining positive for CENP-F were considered in G2 and subsequently analysed. Cells with ≥5 RAD51 foci were deemed RAD51^+^.

### Molecular biology

pLenti CMV Hygro DEST (w117-1) was digested with SalI and BamHI (New England BioLabs, Ipswich, MA, USA) according to manufacturer instructions. *MYC* cDNA was PCR amplified from pcDNA5 FRT/TO CR MYC and cloned into pLenti CMV Hygro DEST, creating pLenti CMV Hygro MYC (Littler et al., 2019). pLVX mCherry N1 (Clontech Laboratories, Mountain View, CA, USA) was digested with XhoI and BamHI (New England BioLabs) according to manufacturer instructions. H2B cDNA was PCR amplified from pcDNA5 FRT/TO GFP-H2B and cloned into pLVX mCherry N1, creating pLVX mCherry-H2B Puro (Morrow et al., 2005). Gibson Assembly was utilized to create lentiGuide-Neo. Briefly, lentiGuide-Puro was PCR amplified, omitting the puromycin-resistance cassette. Separately, the neomycin-resistance cassette was PCR amplified from pLXV MYC-mCherry Neo. Fragments were then assembled into lentiGuide-Neo using Gibson Assembly Master Mix (New England BioLabs) according to manufacturer instructions. gRNAs were introduced into lentiGuide-Puro/Neo by ligating the annealed forward and reverse oligonucleotides into BsmBI-digested target vectors (Sanjana et al., 2014). All recombinant vectors were grown in XL1-Blue competent cells and extracted using QIAprep Spin Miniprep kit (Qiagen, Hilden, Germany) according to manufacturer instructions. Information on oligonucleotide sequences is provided in Table S2. Recombinant vectors were validated functionally *in vitro* or by Sanger sequencing.

### Molecular cytogenetics

For SKY, cells were cultured as normal and incubated in 100 ng/ml Colcemid (Roche, Brighton, MA, USA) for 2 h prior to harvest. Subsequently, for SKY and miFISH, cells were harvested, swelled in hypotonic buffer (0.075 M KCl) for 30 min at 37°C, fixed in methanol/acetic acid (3:1) in three wash steps, dropped onto glass slides and aged for 2 weeks at 37°C. Four probe panels containing five probes each were assembled totalling one centromere probe (CCP10) and 19 gene probes (all custom ordered from CytoTest, Rockville, MD, USA): *COX2* (1q31.1), *PIK3CA* (3q26.32), *FBXW7* (4q31.3), *CCNB1* (5q13.2), *DBC2* (8p21.3), *MYC* (8q24.21), *CDKN2A* (9p21.3), *PTEN* (10q23.31), *CCND1* (11q13.3), *KRAS* (12p12.1), *RB1* (13.14.2), *CDH1* (16q22.1), *TP53* (17p13.1), *NF1* (17q11.2), *HER2* (17q12), *SMAD4* (18q21.2), *CCNE1* (19q12), *ZNF217* (20q13.2) and *NF2* (22q12.2). Images were taken on an automated fluorescence microscope fitted with a 40× oil immersion objective (BX63, Olympus, Tokyo, Japan), custom optical filters (Chroma, Bellows Falls, VT, USA) and a motorized stage. Custom software was used for operation and analysis (BioView, Billerica, MA, USA). A total of 100 nuclei were analysed per sample for miFISH and 15 metaphases were analysed per sample for SKY. Procedures pertaining to SKY and miFISH hybridization, stripping and rehybridization were as described previously (Heselmeyer-Haddad et al., 2012; Padilla-Nash et al., 2006; Wangsa et al., 2018).

### Next-generation sequencing

RNA was extracted from logarithmically growing cells *in situ* using an RNeasy Plus Mini kit (Qiagen) according to manufacturer instructions. RNA integrity and quality were assessed using a 2200 TapeStation (Agilent Technologies, Santa Clara, CA, USA; performed by the CCR Genomics Core, Bethesda, MD, USA). Libraries were prepared using Illumina TruSeq^®^ Stranded mRNA Library Prep (Illumina, San Diego, CA, USA), pooled and paired-end sequenced on Illumina NovaSeq using an SP flow cell according to manufacturer instructions (Sequencing Facility at NCI Frederick, MD, USA). Samples returned 37 to 51 million pass filter reads with more than 91% of bases above the quality score of Q30.

scWGS was performed on single cells sorted for a 2c (parental FNE1, P1,PB1, PB1E, PB1M) or 4c (PB3, PB3E, PB3M) genome content (for PB2, PB2E and PB2M 12 cells from each population were included) as described previously (Bakker et al., 2016; Nelson et al., 2020; van den Bos et al., 2016). FNE1 cells and P1 cells were sequenced at passage 50 and 19, respectively, while PB1/E/M, PB2/E/M and PB3/E/M cells were sequenced between passages 25 and 33.

### Bioinformatics

For RNA sequencing, sample reads were processed using the CCBR Pipeliner utility (https://github.com/CCBR/Pipeliner). Briefly, reads were trimmed for adapters and low-quality bases using Cutadapt (version 1.18) (https://bioweb.pasteur.fr/packages/pack@cutadapt@1.18) before alignment to the human reference genome (hg38/Dec. 2013/GRCh38) from the UCSC browser and the transcripts annotated using STAR v2.4.2a in two-pass mode (Dobin et al., 2013; Martin, 2011). Expression levels were quantified using RSEM (version 1.3.0) (Li and Dewey, 2011) with GENCODE annotation version 30 (Harrow et al., 2012). The same approach was used for mouse model data downloaded from Gene Expression Omnibus (GEO; accession number GSE125016), with alignment to the mouse reference genome (mm10).

Raw read counts (expected counts from RSEM) were imported to the National Institutes of Health Integrated Data Analysis Platform for downstream analysis. Low count genes [counts-per-million (CPM) <0.5], ≥three samples were filtered prior to the analysis. Counts were normalized to library size as CPM, and the voom algorithm (Law et al., 2014) from the Limma R package (version 3.40.6) (Smyth, 2004) was used for quantile normalization (Tables S4 and S7). Batch correction was performed prior to analysis using the ComBat function in the sva package (Johnson et al., 2007). Differentially expressed genes using Limma and pre-ranked GSEA were computed between each genotype using the Molecular Signatures Database (Liberzon et al., 2011; Subramanian et al., 2005). Gene set variation analysis (GSVA) was performed using the GSVA package (Hänzelmann et al., 2013). Genes or gene sets with an adjusted *P*-value ≤0.05 were considered statistically significant. Preparation of heatmaps was performed in R Studio (Subramanian et al., 2005).

Analysis of copy-number changes based on scWGS was executed according to previous reports (Bakker et al., 2016; Nelson et al., 2020; van den Bos et al., 2016).

### Quantification and statistical analysis

Prism 8 (GraphPad, San Diego, CA, USA) was used to generate graphs and perform statistical analyses. RStudio (R Project for Statistical Computing) was used to perform sequencing analyses and generate heatmaps (R packages Complex Heatmaps and AneuFinder).

## Supplementary Material

Supplementary information

## References

[DMM049001C1] Ahmed, A. A., Etemadmoghadam, D., Temple, J., Lynch, A. G., Riad, M., Sharma, R., Stewart, C., Fereday, S., Caldas, C., deFazio, A. et al. (2010). Driver mutations in *TP53* are ubiquitous in high grade serous carcinoma of the ovary. *J. Pathol.* 221, 49-56. 10.1002/path.2696 20229506 PMC3262968

[DMM049001C2] Allen, M. A., Andrysik, Z., Dengler, V. L., Mellert, H. S., Guarnieri, A., Freeman, J. A., Sullivan, K. D., Galbraith, M. D., Luo, X., Kraus, W. L. et al. (2014). Global analysis of p53-regulated transcription identifies its direct targets and unexpected regulatory mechanisms. *eLife* 3, e02200. 10.7554/eLife.02200.019 24867637 PMC4033189

[DMM049001C3] Bakker, B., Taudt, A., Belderbos, M. E., Porubsky, D., Spierings, D. C. J., de Jong, T. V., Halsema, N., Kazemier, H. G., Hoekstra-Wakker, K., Bradley, A. et al. (2016). Single-cell sequencing reveals karyotype heterogeneity in murine and human malignancies. *Genome Biol.* 17, 115. 10.1186/s13059-016-0971-7 27246460 PMC4888588

[DMM049001C4] Barboza, J. A., Liu, G., Ju, Z., El-Naggar, A. K. and Lozano, G. (2006). p21 delays tumor onset by preservation of chromosomal stability. *Proc. Natl. Acad. Sci. USA* 103, 19842-19847. 10.1073/pnas.0606343104 17170138 PMC1702317

[DMM049001C5] Bastians, H. (2015). Causes of chromosomal instability. *Recent Results Cancer Res.* 200, 95-113. 10.1007/978-3-319-20291-4_5 26376874

[DMM049001C6] Bennett, A., Bechi, B., Tighe, A., Thompson, S., Procter, D. J. and Taylor, S. S. (2015). Cenp-E inhibitor GSK923295: Novel synthetic route and use as a tool to generate aneuploidy. *Oncotarget* 6, 20921-20932. 10.18632/oncotarget.4879 26320186 PMC4673239

[DMM049001C7] Bianco, J. N., Bergoglio, V., Lin, Y.-L., Pillaire, M.-J., Schmitz, A.-L., Gilhodes, J., Lusque, A., Mazières, J., Lacroix-Triki, M., Roumeliotis, T. I. et al. (2019). Overexpression of Claspin and Timeless protects cancer cells from replication stress in a checkpoint-independent manner. *Nat. Commun.* 10, 910. 10.1038/s41467-019-08886-8 30796221 PMC6385232

[DMM049001C8] Bielski, C. M., Zehir, A., Penson, A. V., Donoghue, M. T. A., Chatila, W., Armenia, J., Chang, M. T., Schram, A. M., Jonsson, P., Bandlamudi, C. et al. (2018). Genome doubling shapes the evolution and prognosis of advanced cancers. *Nat. Genet.* 50, 1189-1195. 10.1038/s41588-018-0165-1 30013179 PMC6072608

[DMM049001C9] Bowtell, D. D., Böhm, S., Ahmed, A. A., Aspuria, P.-J., Bast, R. C., Jr, Beral, V., Berek, J. S., Birrer, M. J., Blagden, S., Bookman, M. A. et al. (2015). Rethinking ovarian cancer II: reducing mortality from high-grade serous ovarian cancer. *Nat. Rev. Cancer* 15, 668-679. 10.1038/nrc4019 26493647 PMC4892184

[DMM049001C10] Bunz, F., Fauth, C., Speicher, M. R., Dutriaux, A., Sedivy, J. M., Kinzler, K. W., Vogelstein, B. and Lengauer, C. (2002). Targeted inactivation of p53 in human cells does not result in aneuploidy. *Cancer Res.* 62, 1129-1133. 11861393

[DMM049001C11] Burrell, R. A., McClelland, S. E., Endesfelder, D., Groth, P., Weller, M.-C., Shaikh, N., Domingo, E., Kanu, N., Dewhurst, S. M., Gronroos, E. et al. (2013). Replication stress links structural and numerical cancer chromosomal instability. *Nature* 494, 492-496. 10.1038/nature11935 23446422 PMC4636055

[DMM049001C12] Campeau, E., Ruhl, V. E., Rodier, F., Smith, C. L., Rahmberg, B. L., Fuss, J. O., Campisi, J., Yaswen, P., Cooper, P. K. and Kaufman, P. D. (2009). A versatile viral system for expression and depletion of proteins in mammalian cells. *PLoS ONE* 4, e6529. 10.1371/journal.pone.0006529 19657394 PMC2717805

[DMM049001C13] Cancer Genome Atlas Research Network. (2011). Integrated genomic analyses of ovarian carcinoma. *Nature* 474, 609-615. 10.1038/nature10166 21720365 PMC3163504

[DMM049001C14] Chui, M. H., Doodnauth, S. A., Erdmann, N., Tiedemann, R. E., Sircoulomb, F., Drapkin, R., Shaw, P. and Rottapel, R. (2019). Chromosomal instability and mTORC1 activation through PTEN loss contribute to proteotoxic stress in ovarian carcinoma. *Cancer Res.* 79, 5536-5549. 10.1158/0008-5472.CAN-18-3029 31530568

[DMM049001C15] Ciriello, G., Miller, M. L., Aksoy, B. A., Senbabaoglu, Y., Schultz, N. and Sander, C. (2013). Emerging landscape of oncogenic signatures across human cancers. *Nat. Genet.* 45, 1127-1133. 10.1038/ng.2762 24071851 PMC4320046

[DMM049001C16] Davoli, T., Uno, H., Wooten, E. C. and Elledge, S. J. (2017). Tumor aneuploidy correlates with markers of immune evasion and with reduced response to immunotherapy. *Science* 355, eaaf8399. 10.1126/science.aaf8399 28104840 PMC5592794

[DMM049001C17] Di Paolo, A., Racca, C., Calsou, P. and Larminat, F. (2014). Loss of BRCA1 impairs centromeric cohesion and triggers chromosomal instability. *FASEB J.* 28, 5250-5261. 10.1096/fj.14-250266 25205741

[DMM049001C18] Dobin, A., Davis, C. A., Schlesinger, F., Drenkow, J., Zaleski, C., Jha, S., Batut, P., Chaisson, M. and Gingeras, T. R. (2013). STAR: ultrafast universal RNA-seq aligner. *Bioinformatics* 29, 15-21. 10.1093/bioinformatics/bts635 23104886 PMC3530905

[DMM049001C19] Ducie, J., Dao, F., Considine, M., Olvera, N., Shaw, P. A., Kurman, R. J., Shih, I.-M., Soslow, R. A., Cope, L. and Levine, D. A. (2017). Molecular analysis of high-grade serous ovarian carcinoma with and without associated serous tubal intra-epithelial carcinoma. *Nat. Commun.* 8, 990. 10.1038/s41467-017-01217-9 29042553 PMC5645359

[DMM049001C20] Fujiwara, T., Bandi, M., Nitta, M., Ivanova, E. V., Bronson, R. T. and Pellman, D. (2005). Cytokinesis failure generating tetraploids promotes tumorigenesis in p53-null cells. *Nature* 437, 1043-1047. 10.1038/nature04217 16222300

[DMM049001C21] Funnell, T., O'Flanagan, C. H., Williams, M. J., McPherson, A., McKinney, S., Kabeer, F., Lee, H., Masud, T., Eirew, P., Yap, D. et al. (2021). The impact of mutational processes on structural genomic plasticity in cancer cells. *bioRxiv*, 2021.2006.2003.446999.

[DMM049001C22] Hänzelmann, S., Castelo, R. and Guinney, J. (2013). GSVA: gene set variation analysis for microarray and RNA-seq data. *BMC Bioinformatics* 14, 7. 10.1186/1471-2105-14-7 23323831 PMC3618321

[DMM049001C23] Harrow, J., Frankish, A., Gonzalez, J. M., Tapanari, E., Diekhans, M., Kokocinski, F., Aken, B. L., Barrell, D., Zadissa, A., Searle, S. et al. (2012). GENCODE: The reference human genome annotation for The ENCODE Project. *Genome Res.* 22, 1760-1774. 10.1101/gr.135350.111 22955987 PMC3431492

[DMM049001C24] Heselmeyer-Haddad, K., Berroa Garcia, L. Y., Bradley, A., Ortiz-Melendez, C., Lee, W.-J., Christensen, R., Prindiville, S. A., Calzone, K. A., Soballe, P. W., Hu, Y. et al. (2012). Single-cell genetic analysis of ductal carcinoma *in situ* and invasive breast cancer reveals enormous tumor heterogeneity yet conserved genomic imbalances and gain of *MYC* during progression. *Am. J. Pathol.* 181, 1807-1822. 10.1016/j.ajpath.2012.07.012 23062488 PMC3483801

[DMM049001C25] Huschtscha, L. I. and Reddel, R. R. (1999). p16(INK4a) and the control of cellular proliferative life span. *Carcinogenesis* 20, 921-926. 10.1093/carcin/20.6.921 10357768

[DMM049001C26] Hussein, D. and Taylor, S. S. (2002). Farnesylation of Cenp-F is required for G2/M progression and degradation after mitosis. *J. Cell Sci.* 115, 3403-3414. 10.1242/jcs.115.17.3403 12154071

[DMM049001C27] Jiménez-Sánchez, A., Cybulska, P., Mager, K. L. V., Koplev, S., Cast, O., Couturier, D.-L., Memon, D., Selenica, P., Nikolovski, I., Mazaheri, Y. et al. (2020). Unraveling tumor-immune heterogeneity in advanced ovarian cancer uncovers immunogenic effect of chemotherapy. *Nat. Genet.* 52, 582-593. 10.1038/s41588-020-0630-5 32483290 PMC8353209

[DMM049001C28] Johnson, W. E., Li, C. and Rabinovic, A. (2007). Adjusting batch effects in microarray expression data using empirical Bayes methods. *Biostatistics* 8, 118-127. 10.1093/biostatistics/kxj037 16632515

[DMM049001C29] Karst, A. M. and Drapkin, R. (2012). Primary culture and immortalization of human fallopian tube secretory epithelial cells. *Nat. Protoc.* 7, 1755-1764. 10.1038/nprot.2012.097 22936217 PMC7433321

[DMM049001C30] Karst, A. M., Levanon, K. and Drapkin, R. (2011). Modeling high-grade serous ovarian carcinogenesis from the fallopian tube. *Proc. Natl. Acad. Sci. USA* 108, 7547-7552. 10.1073/pnas.1017300108 21502498 PMC3088633

[DMM049001C31] Kok, Y. P., Guerrero Llobet, S., Schoonen, P. M., Everts, M., Bhattacharya, A., Fehrmann, R. S. N., van den Tempel, N. and van Vugt, M. A. T. M. (2020). Overexpression of Cyclin E1 or Cdc25A leads to replication stress, mitotic aberrancies, and increased sensitivity to replication checkpoint inhibitors. *Oncogenesis* 9, 88. 10.1038/s41389-020-00270-2 33028815 PMC7542455

[DMM049001C32] Kotsantis, P., Petermann, E. and Boulton, S. J. (2018). Mechanisms of oncogene-induced replication stress: jigsaw falling into place. *Cancer Discov.* 8, 537-555. 10.1158/2159-8290.CD-17-1461 29653955 PMC5935233

[DMM049001C33] Kuznetsova, A. Y., Seget, K., Moeller, G. K., de Pagter, M. S., de Roos, J. A. D. M., Dürrbaum, M., Kuffer, C., Müller, S., Zaman, G. J. R., Kloosterman, W. P. et al. (2015). Chromosomal instability, tolerance of mitotic errors and multidrug resistance are promoted by tetraploidization in human cells. *Cell Cycle* 14, 2810-2820. 10.1080/15384101.2015.1068482 26151317 PMC4614355

[DMM049001C34] Labidi-Galy, S. I., Papp, E., Hallberg, D., Niknafs, N., Adleff, V., Noe, M., Bhattacharya, R., Novak, M., Jones, S., Phallen, J. et al. (2017). High grade serous ovarian carcinomas originate in the fallopian tube. *Nat. Commun.* 8, 1093. 10.1038/s41467-017-00962-1 29061967 PMC5653668

[DMM049001C35] Lambrus, B. G., Daggubati, V., Uetake, Y., Scott, P. M., Clutario, K. M., Sluder, G. and Holland, A. J. (2016). A USP28-53BP1-p53-p21 signaling axis arrests growth after centrosome loss or prolonged mitosis. *J. Cell Biol.* 214, 143-153. 10.1083/jcb.201604054 27432896 PMC4949452

[DMM049001C36] Law, C. W., Chen, Y., Shi, W. and Smyth, G. K. (2014). voom: precision weights unlock linear model analysis tools for RNA-seq read counts. *Genome Biol.* 15, R29. 10.1186/gb-2014-15-2-r29 24485249 PMC4053721

[DMM049001C37] Li, B. and Dewey, C. N. (2011). RSEM: accurate transcript quantification from RNA-Seq data with or without a reference genome. *BMC Bioinformatics* 12, 323. 10.1186/1471-2105-12-323 21816040 PMC3163565

[DMM049001C38] Liberzon, A., Subramanian, A., Pinchback, R., Thorvaldsdóttir, H., Tamayo, P. and Mesirov, J. P. (2011). Molecular signatures database (MSigDB) 3.0. *Bioinformatics* 27, 1739-1740. 10.1093/bioinformatics/btr260 21546393 PMC3106198

[DMM049001C39] Lin, C. Y., Lovén, J., Rahl, P. B., Paranal, R. M., Burge, C. B., Bradner, J. E., Lee, T. I. and Young, R. A. (2012). Transcriptional amplification in tumor cells with elevated c-Myc. *Cell* 151, 56-67. 10.1016/j.cell.2012.08.026 23021215 PMC3462372

[DMM049001C40] Littler, S., Sloss, O., Geary, B., Pierce, A., Whetton, A. D. and Taylor, S. S. (2019). Oncogenic MYC amplifies mitotic perturbations. *Open Biol* 9, 190136. 10.1098/rsob.190136 31455158 PMC6731591

[DMM049001C41] Macintyre, G., Goranova, T. E., De Silva, D., Ennis, D., Piskorz, A. M., Eldridge, M., Sie, D., Lewsley, L.-A., Hanif, A., Wilson, C. et al. (2018). Copy number signatures and mutational processes in ovarian carcinoma. *Nat. Genet.* 50, 1262-1270. 10.1038/s41588-018-0179-8 30104763 PMC6130818

[DMM049001C42] Martin, M. (2011). Cutadapt removes adapter sequences from high-throughput sequencing reads. *EMBnet J.* 17, 3. 10.14806/ej.17.1.200

[DMM049001C43] McDermott, J. E., Arshad, O. A., Petyuk, V. A., Fu, Y., Gritsenko, M. A., Clauss, T. R., Moore, R. J., Schepmoes, A. A., Zhao, R., Monroe, M. E. et al. (2020). Proteogenomic characterization of ovarian HGSC implicates mitotic kinases, replication stress in observed chromosomal instability. *Cell Rep. Med.* 1, 100075. 10.1016/j.xcrm.2020.100075 32529193 PMC7289043

[DMM049001C44] Merritt, M. A., Bentink, S., Schwede, M., Iwanicki, M. P., Quackenbush, J., Woo, T., Agoston, E. S., Reinhardt, F., Crum, C. P., Berkowitz, R. S. et al. (2013). Gene expression signature of normal cell-of-origin predicts ovarian tumor outcomes. *PLoS ONE* 8, e80314. 10.1371/journal.pone.0080314 24303006 PMC3841174

[DMM049001C45] Morrow, C. J., Tighe, A., Johnson, V. L., Scott, M. I. F., Ditchfield, C. and Taylor, S. S. (2005). Bub1 and aurora B cooperate to maintain BubR1-mediated inhibition of APC/CCdc20. *J. Cell Sci.* 118, 3639-3652. 10.1242/jcs.02487 16046481

[DMM049001C46] Nakamura, K., Nakayama, K., Ishikawa, N., Ishikawa, M., Sultana, R., Kiyono, T. and Kyo, S. (2018). Reconstitution of high-grade serous ovarian carcinoma from primary fallopian tube secretory epithelial cells. *Oncotarget* 9, 12609-12619. 10.18632/oncotarget.23035 29560094 PMC5849158

[DMM049001C47] Nelson, L., Tighe, A., Golder, A., Littler, S., Bakker, B., Moralli, D., Murtuza Baker, S., Donaldson, I. J., Spierings, D. C. J., Wardenaar, R. et al. (2020). A living biobank of ovarian cancer ex vivo models reveals profound mitotic heterogeneity. *Nat. Commun.* 11, 822. 10.1038/s41467-020-14551-2 32054838 PMC7018727

[DMM049001C48] Nie, Z., Hu, G., Wei, G., Cui, K., Yamane, A., Resch, W., Wang, R., Green, D. R., Tessarollo, L., Casellas, R. et al. (2012). c-Myc is a universal amplifier of expressed genes in lymphocytes and embryonic stem cells. *Cell* 151, 68-79. 10.1016/j.cell.2012.08.033 23021216 PMC3471363

[DMM049001C49] Nie, Z., Guo, C., Das, S. K., Chow, C. C., Batchelor, E., Simons, S. S., Jr and Levens, D. (2020). Dissecting transcriptional amplification by MYC. *eLife* 9, e52483. 10.7554/eLife.52483 32715994 PMC7384857

[DMM049001C50] Ogino, S., Gulley, M. L., den Dunnen, J.T., Wilson, R. B. and the Association for Molecular Pathology Training and Education Committee. (2007). Standard mutation nomenclature in molecular diagnostics: practical and educational challenges. *J. Mol. Diagn.* 9, 1-6. 10.2353/jmoldx.2007.060081 17251329 PMC1867422

[DMM049001C51] Padilla-Nash, H. M., Barenboim-Stapleton, L., Difilippantonio, M. J. and Ried, T. (2006). Spectral karyotyping analysis of human and mouse chromosomes. *Nat. Protoc.* 1, 3129-3142. 10.1038/nprot.2006.358 17406576 PMC4772431

[DMM049001C52] Robinson, J. T., Thorvaldsdóttir, H., Winckler, W., Guttman, M., Lander, E. S., Getz, G. and Mesirov, J. P. (2011). Integrative genomics viewer. *Nat. Biotechnol.* 29, 24-26. 10.1038/nbt.1754 21221095 PMC3346182

[DMM049001C53] Rohrberg, J., Van de Mark, D., Amouzgar, M., Lee, J. V., Taileb, M., Corella, A., Kilinc, S., Williams, J., Jokisch, M.-L., Camarda, R. et al. (2020). MYC dysregulates mitosis, revealing cancer vulnerabilities. *Cell Rep.* 30, 3368-3382.e3367. 10.1016/j.celrep.2020.02.041 32160543 PMC7085414

[DMM049001C54] Rutledge, S. D., Douglas, T. A., Nicholson, J. M., Vila-Casadesús, M., Kantzler, C. L., Wangsa, D., Barroso-Vilares, M., Kale, S. D., Logarinho, E. and Cimini, D. (2016). Selective advantage of trisomic human cells cultured in non-standard conditions. *Sci. Rep.* 6, 22828. 10.1038/srep22828 26956415 PMC4783771

[DMM049001C55] Salehi, S., Kabeer, F., Ceglia, N., Andronescu, M., Williams, M. J., Campbell, K. R., Masud, T., Wang, B., Biele, J., Brimhall, J. et al. (2021). Clonal fitness inferred from time-series modelling of single-cell cancer genomes. *Nature* 595, 585-590. 10.1038/s41586-021-03648-3 34163070 PMC8396073

[DMM049001C56] Sanjana, N. E., Shalem, O. and Zhang, F. (2014). Improved vectors and genome-wide libraries for CRISPR screening. *Nat. Methods* 11, 783-784. 10.1038/nmeth.3047 25075903 PMC4486245

[DMM049001C57] Santaguida, S., Richardson, A., Iyer, D. R., M'Saad, O., Zasadil, L., Knouse, K. A., Wong, Y. L., Rhind, N., Desai, A. and Amon, A. (2017). Chromosome mis-segregation generates cell-cycle-arrested cells with complex karyotypes that are eliminated by the immune system. *Dev. Cell* 41, 638-651.e635. 10.1016/j.devcel.2017.05.022 28633018 PMC5536848

[DMM049001C58] Shukla, A., Nguyen, T. H. M., Moka, S. B., Ellis, J. J., Grady, J. P., Oey, H., Cristino, A. S., Khanna, K. K., Kroese, D. P., Krause, L. et al. (2020). Chromosome arm aneuploidies shape tumour evolution and drug response. *Nat. Commun.* 11, 449. 10.1038/s41467-020-14286-0 31974379 PMC6978319

[DMM049001C59] Simões-Sousa, S., Littler, S., Thompson, S. L., Minshall, P., Whalley, H., Bakker, B., Belkot, K., Moralli, D., Bronder, D., Tighe, A. et al. (2018). The p38α stress kinase suppresses aneuploidy tolerance by inhibiting Hif-1α. *Cell Rep.* 25, 749-760.e746. 10.1016/j.celrep.2018.09.060 30332653 PMC6205844

[DMM049001C60] Smyth, G. K. (2004). Linear models and empirical bayes methods for assessing differential expression in microarray experiments. *Stat. Appl. Genet. Mol. Biol.* 3, Article3. 10.2202/1544-6115.1027 16646809

[DMM049001C61] Soto, M., Raaijmakers, J. A., Bakker, B., Spierings, D. C. J., Lansdorp, P. M., Foijer, F. and Medema, R. H. (2017). p53 prohibits propagation of chromosome segregation errors that produce structural aneuploidies. *Cell Rep.* 19, 2423-2431. 10.1016/j.celrep.2017.05.055 28636931

[DMM049001C62] Subramanian, A., Tamayo, P., Mootha, V. K., Mukherjee, S., Ebert, B. L., Gillette, M. A., Paulovich, A., Pomeroy, S. L., Golub, T. R., Lander, E. S. et al. (2005). Gene set enrichment analysis: a knowledge-based approach for interpreting genome-wide expression profiles. *Proc. Natl. Acad. Sci. USA* 102, 15545-15550. 10.1073/pnas.0506580102 16199517 PMC1239896

[DMM049001C63] Sullivan, K. D., Galbraith, M. D., Andrysik, Z. and Espinosa, J. M. (2018). Mechanisms of transcriptional regulation by p53. *Cell Death Differ.* 25, 133-143. 10.1038/cdd.2017.174 29125602 PMC5729533

[DMM049001C64] Tamura, N., Shaikh, N., Muliaditan, D., Soliman, T. N., McGuinness, J. R., Maniati, E., Moralli, D., Durin, M. A., Green, C. M., Balkwill, F. R. et al. (2020). Specific mechanisms of chromosomal instability indicate therapeutic sensitivities in high-grade serous ovarian carcinoma. *Cancer Res.* 80, 4946-4959. 10.1158/0008-5472.CAN-19-0852 32998996

[DMM049001C65] Taylor, A. M., Shih, J., Ha, G., Gao, G. F., Zhang, X., Berger, A. C., Schumacher, S. E., Wang, C., Hu, H., Liu, J. et al. (2018). Genomic and functional approaches to understanding cancer aneuploidy. *Cancer Cell* 33, 676-689.e673. 10.1016/j.ccell.2018.03.007 29622463 PMC6028190

[DMM049001C66] Thompson, S. L. and Compton, D. A. (2010). Proliferation of aneuploid human cells is limited by a p53-dependent mechanism. *J. Cell Biol.* 188, 369-381. 10.1083/jcb.200905057 20123995 PMC2819684

[DMM049001C67] Valente, L. J., Tarangelo, A., Li, A. M., Naciri, M., Raj, N., Boutelle, A. M., Li, Y., Mello, S. S., Bieging-Rolett, K., DeBerardinis, R. J. et al. (2020). p53 deficiency triggers dysregulation of diverse cellular processes in physiological oxygen. *J. Cell Biol.* 219, e201908212. 10.1083/jcb.201908212 32886745 PMC7594498

[DMM049001C68] van den Bos, H., Spierings, D. C. J., Taudt, A., Bakker, B., Porubský, D., Falconer, E., Novoa, C., Halsema, N., Kazemier, H. G., Hoekstra-Wakker, K. et al. (2016). Single-cell whole genome sequencing reveals no evidence for common aneuploidy in normal and Alzheimer's disease neurons. *Genome Biol.* 17, 116. 10.1186/s13059-016-0976-2 27246599 PMC4888403

[DMM049001C69] Vang, R., Levine, D. A., Soslow, R. A., Zaloudek, C., Shih, I.-M. and Kurman, R. J. (2016). Molecular alterations of TP53 are a defining feature of ovarian high-grade serous carcinoma: a rereview of cases lacking TP53 mutations in The Cancer Genome Atlas Ovarian Study. *Int. J. Gynecol. Pathol.* 35, 48-55. 10.1097/PGP.0000000000000207 26166714 PMC4696053

[DMM049001C70] Vassilev, L. T., Vu, B. T., Graves, B., Carvajal, D., Podlaski, F., Filipovic, Z., Kong, N., Kammlott, U., Lukacs, C., Klein, C. et al. (2004). In vivo activation of the p53 pathway by small-molecule antagonists of MDM2. *Science* 303, 844-848. 10.1126/science.1092472 14704432

[DMM049001C71] Wang, Y., Bernhardy, A. J., Cruz, C., Krais, J. J., Nacson, J., Nicolas, E., Peri, S., van der Gulden, H., van der Heijden, I., O'Brien, S. W. et al. (2016). The BRCA1-Δ11q alternative splice isoform bypasses germline mutations and promotes therapeutic resistance to PARP inhibition and Cisplatin. *Cancer Res.* 76, 2778-2790. 10.1158/0008-5472.CAN-16-0186 27197267 PMC4874568

[DMM049001C72] Wang, Y. K., Bashashati, A., Anglesio, M. S., Cochrane, D. R., Grewal, D. S., Ha, G., McPherson, A., Horlings, H. M., Senz, J., Prentice, L. M. et al. (2017). Genomic consequences of aberrant DNA repair mechanisms stratify ovarian cancer histotypes. *Nat. Genet.* 49, 856-865. 10.1038/ng.3849 28436987

[DMM049001C73] Wangsa, D., Braun, R., Schiefer, M., Gertz, E. M., Bronder, D., Quintanilla, I., Padilla-Nash, H. M., Torres, I., Hunn, C., Warner, L. et al. (2018). The evolution of single cell-derived colorectal cancer cell lines is dominated by the continued selection of tumor-specific genomic imbalances, despite random chromosomal instability. *Carcinogenesis* 39, 993-1005. 10.1093/carcin/bgy068 29800151 PMC6067130

[DMM049001C74] Weaver, Z., Montagna, C., Xu, X., Howard, T., Gadina, M., Brodie, S. G., Deng, C.-X. and Ried, T. (2002). Mammary tumors in mice conditionally mutant for *Brca1* exhibit gross genomic instability and centrosome amplification yet display a recurring distribution of genomic imbalances that is similar to human breast cancer. *Oncogene* 21, 5097-5107. 10.1038/sj.onc.1205636 12140760

[DMM049001C75] Wood, K. W., Lad, L., Luo, L., Qian, X., Knight, S. D., Nevins, N., Brejc, K., Sutton, D., Gilmartin, A. G., Chua, P. R. et al. (2010). Antitumor activity of an allosteric inhibitor of centromere-associated protein-E. *Proc. Natl. Acad. Sci. USA* 107, 5839-5844. 10.1073/pnas.0915068107 20167803 PMC2851928

[DMM049001C76] Xu, X., Weaver, Z., Linke, S. P., Li, C., Gotay, J., Wang, X.-W., Harris, C. C., Ried, T. and Deng, C.-X. (1999). Centrosome amplification and a defective G2-M cell cycle checkpoint induce genetic instability in BRCA1 exon 11 isoform-deficient cells. *Mol. Cell* 3, 389-395. 10.1016/S1097-2765(00)80466-9 10198641

[DMM049001C77] Yu, V. M., Marion, C. M., Austria, T. M., Yeh, J., Schönthal, A. H. and Dubeau, L. (2012). Role of BRCA1 in controlling mitotic arrest in ovarian cystadenoma cells. *Int. J. Cancer* 130, 2495-2504. 10.1002/ijc.26309 21792894 PMC3299830

[DMM049001C78] Zack, T. I., Schumacher, S. E., Carter, S. L., Cherniack, A. D., Saksena, G., Tabak, B., Lawrence, M. S., Zhang, C.-Z., Wala, J., Mermel, C. H. et al. (2013). Pan-cancer patterns of somatic copy number alteration. *Nat. Genet.* 45, 1134-1140. 10.1038/ng.2760 24071852 PMC3966983

[DMM049001C79] Zeng, M., Kwiatkowski, N. P., Zhang, T., Nabet, B., Xu, M., Liang, Y., Quan, C., Wang, J., Hao, M., Palakurthi, S. et al. (2018). Targeting MYC dependency in ovarian cancer through inhibition of CDK7 and CDK12/13. *eLife* 7, e39030. 10.7554/eLife.39030 30422115 PMC6251623

[DMM049001C80] Zhang, S., Dolgalev, I., Zhang, T., Ran, H., Levine, D. A. and Neel, B. G. (2019). Both fallopian tube and ovarian surface epithelium are cells-of-origin for high-grade serous ovarian carcinoma. *Nat. Commun.* 10, 5367. 10.1038/s41467-019-13116-2 31772167 PMC6879755

