# Supplementary material for: TP53 loss initiates chromosomal instability in fallopian tube epithelial cells
Source: Dis Model Mech. 2021 Nov 30;14(11):dmm049001. doi: 10.1242/dmm.049001 (PMC8649171; doi:10.1242/dmm.049001)
Supplement: Supplementary information [file dmm-14-049001-s1.pdf]

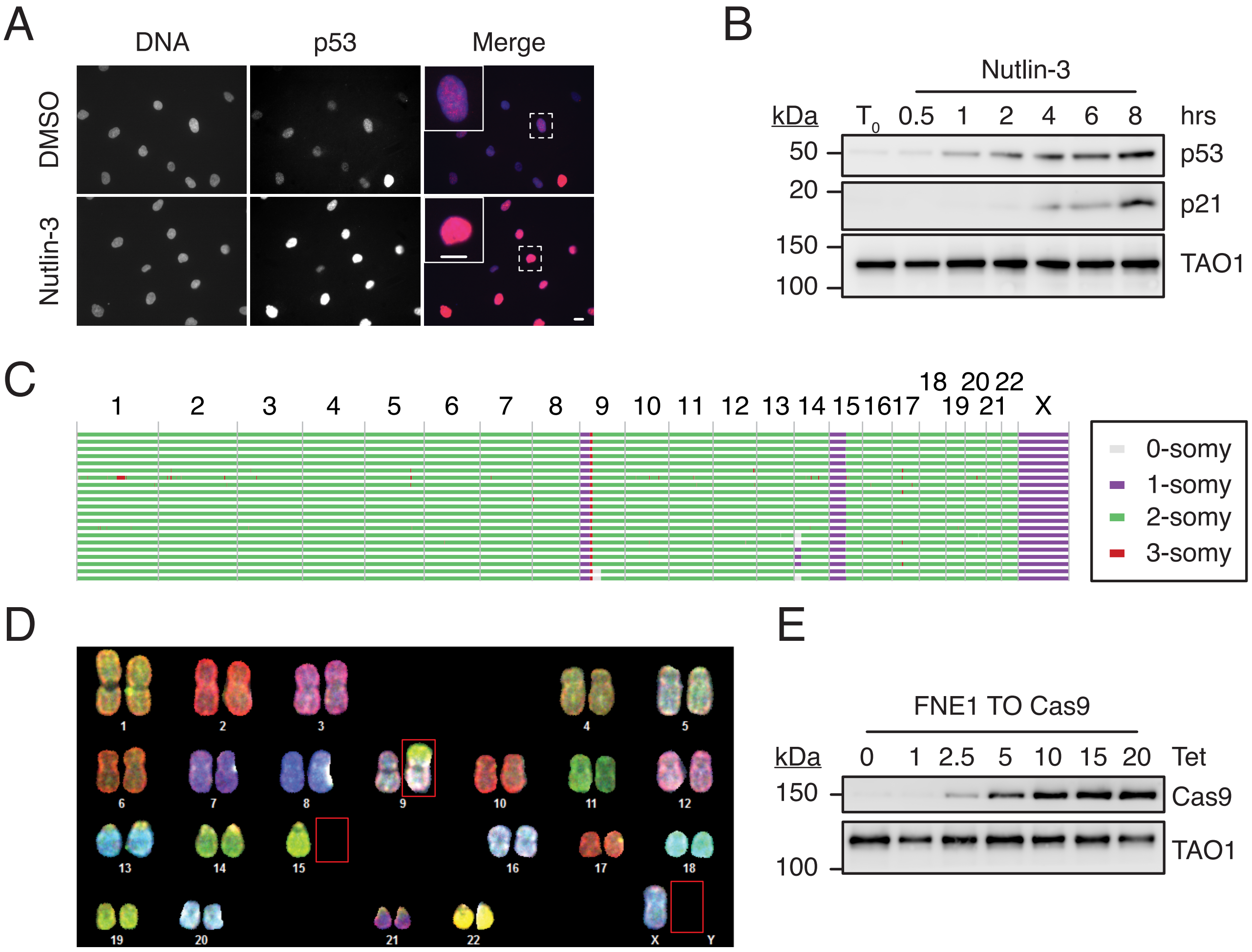

Disease Models & Mechanisms • Supplementary information

Fig. S1. FNE1 Characterization

A Immunofluorescence imaging of DMSO (vehicle) and Nutlin-3-treated parental FNE1 cells shows stabilization of p53 in response to Nutlin-3. Representative images from one of three experiments. Scale bars, 10 µm.

B Immunoblot of cells treated with Nutlin-3 over a time course of 8 hours to analyse p53 and p21 expression. TAO1 serves as loading control.

C Shallow-depth, whole-genome sequencing analysis of copy number aberrations in single parental FNE1 cells (rows) where columns reflect chromosomes 1–22 and X. Colour indicates detected copy number level (box).

D Spectral karyotyping image of a representative metaphase spread shows a near-diploid genome with loss of chromosomes 15 and X and translocation between 9p and 15q (red boxes).

E Immunoblot of tet-inducible Cas9 expression in parental FNE1 cells after transduction with Edit-R Inducible Lentiviral Cas9 and selection. Subsequent experiments utilized 15 µg ml^{-1} tet for Cas9 induction. TAO1 serves as loading control. Tet=µg ml^{-1} tetracycline.

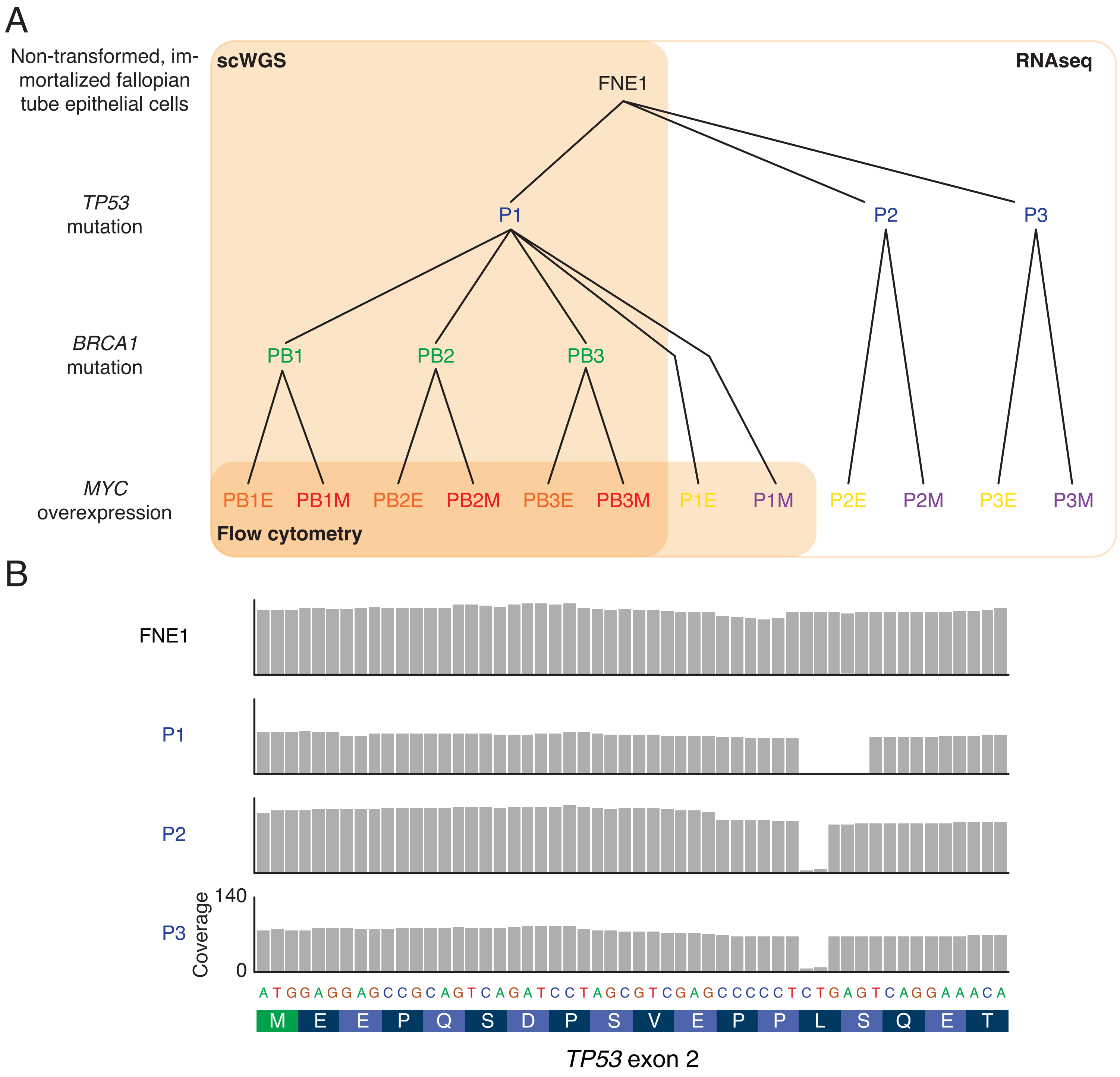

Disease Models & Mechanisms • Supplementary information

Fig. S2. Pedigree of Mutant Subclones and *TP53* Locus Mutation

A Pedigree of FNE1 cells and sequentially CRISPR/Cas9-mediated genome-engineered subclones with introduction of MYC overexpression or empty-lentiviral construct.

B Coverage of RNA sequencing reads of *TP53* exon 2 in indicated subclones. Deletion of 2–5 nucleotides in the three mutagenized subclones is shown, resulting in a downstream premature termination codon.

P=*TP53*-mutant; B=*BRCA1*-mutant; E=empty-vector lentivirus; M=*MYC*-overexpressing lentivirus.

Disease Models & Mechanisms • Supplementary information

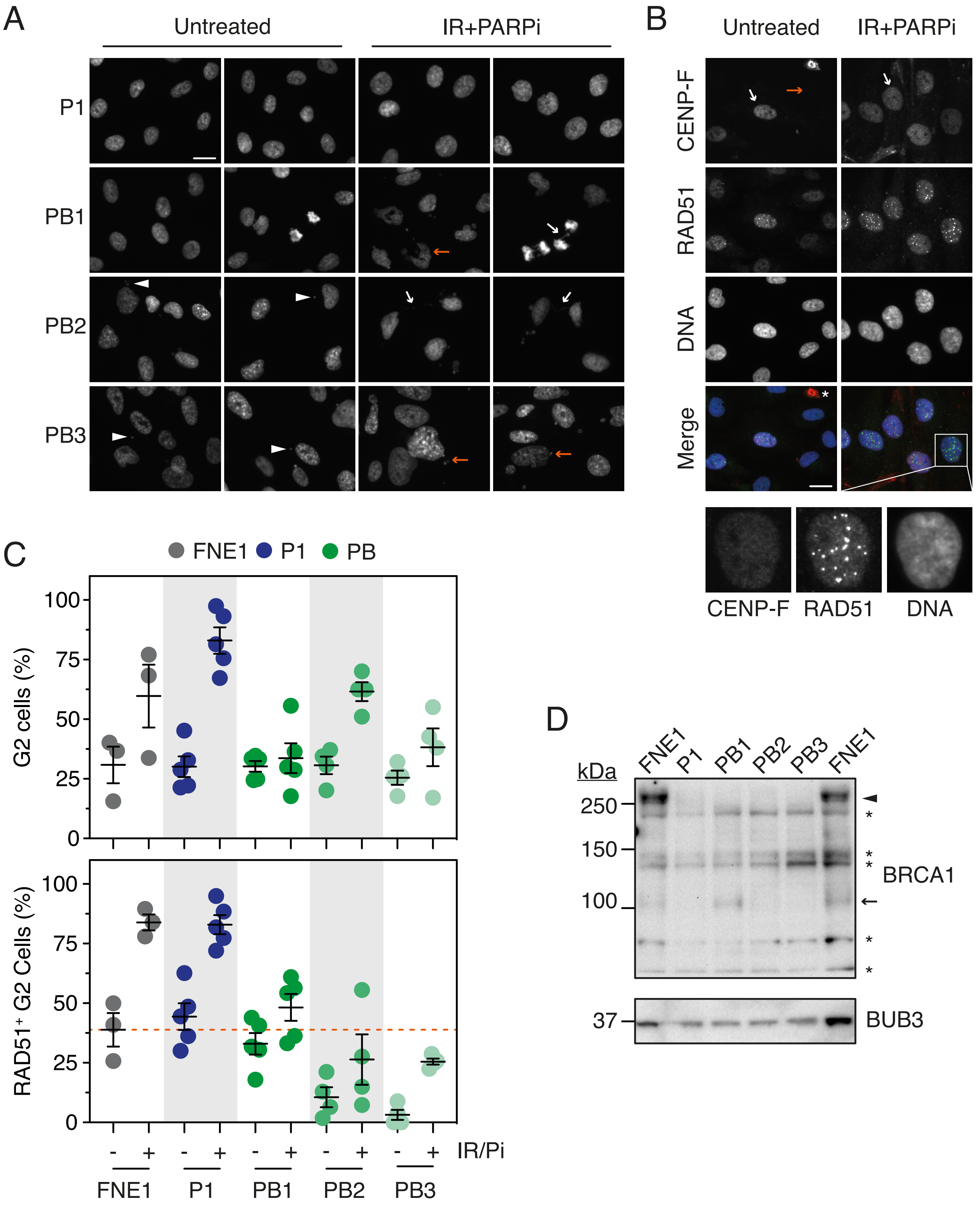

Disease Models & Mechanisms • Supplementary information

Fig. S3. BRCA1 status of PB lineages

A Immunofluorescence images showing nuclei in parental P1 cells and the three BRCA1-mutant PB lineages, PB1, PB2 and PB3. Cells were either untreated or exposed to 2 Gy x-ray and 1 µM PARPi Olaparib before being fixed and stained 24 hours later. Arrowheads identify micronuclei in untreated PB2 and PB3 cells. White and orange arrows show chromatin bridges and micronuclei respectively in treated cells. Scale bar, 20 µm.

B Immunofluorescence images of P1 cells, either untreated or exposed to 2 Gy x-ray and 1 µM PARPi Olaparib stained to detect CENP-F (red), RAD51 (green) and DNA (blue), 24 hours following treatment. The orange arrow highlights a CENP-F negative cell in the untreated population while the white arrows identify CENP-F-positive cells. White asterisk, background particle. Scale bar, 20 µm. Inset shows enlargement to identify RAD51 foci.

C Scatter plots quantitating the percentage of G2 cells, i.e., the number of CENP-F positive cells as a percentage of total cell number (upper panel) and the percentage of RAD51 positive cells as a percentage of G2 cells (lower panel). FNE1, P1, PB1, PB2 and PB3 cells were analysed 24 hours after exposure to 2 Gy x-ray and 1 µM PARPi Olaparib. Results from at least three independent experiments are shown, error bars represent standard error of the mean. Note, these data are reproduced in Fig. 2D.

D Immunoblot probed to detect BRCA1 and BUB3 in total cell lysates prepared from FNE1, P1, PB1, PB2 and PB3 cells. Arrowhead identifies full length BRCA1, the arrow identifies the splicing variant in parental FNE1 and PB1 cells, asterisks identify cross reacting proteins. BUB3 is used as loading control. Note, due to technical difficulties we failed to detect BRCA1 in P1 cells, which is shown in Fig. 2C. P=*TP53*-mutant; B=*BRCA1*-mutant.

Disease Models & Mechanisms • Supplementary information

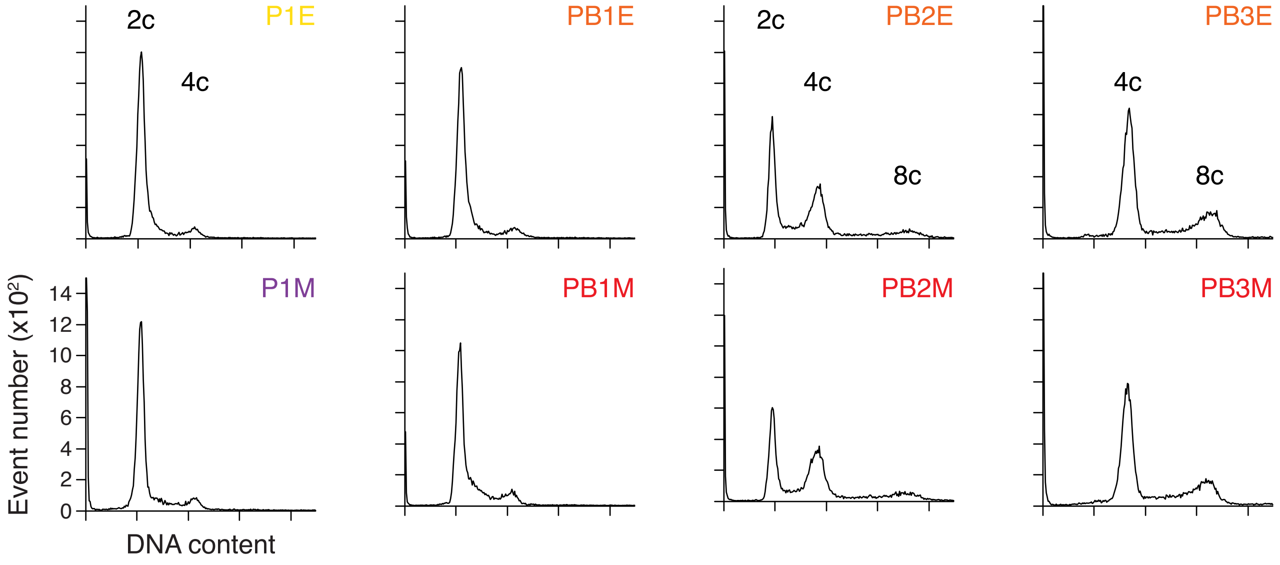

Fig. S4. Genome Content of PB2 and PB3 Clones Suggests Aneuploidy Flow cytometric analysis of genome content in control (empty-vector) and MYC overexpressing cells of the same genotype. 2c, 4c and 8c correspond to a diploid, tetraploid and octoploid genome. P=*TP53*-mutant; B=*BRCA1*-mutant; E=empty-vector lentivirus; M=*MYC*-overexpressing lentivirus.

Disease Models & Mechanisms • Supplementary information

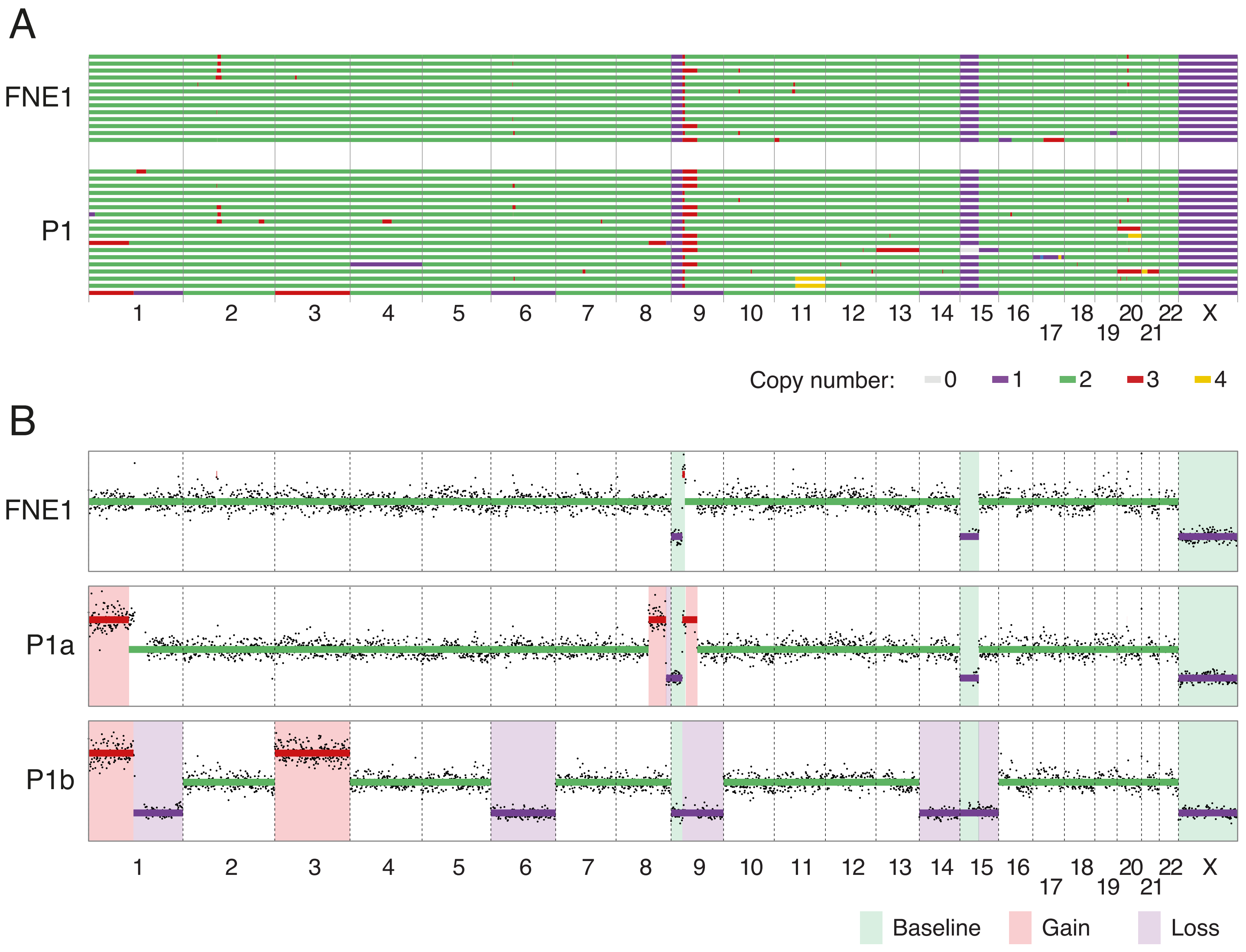

Fig. S5. Aneuploidy Increases Following Loss of p53 in FNE1 Cells

A Shallow-depth, whole-genome sequencing analysis of copy number aberrations in single cells (rows). FNE1_1 and P1 cells are shown on top and bottom, respectively. Columns reflect chromosomes 1–22 and X. Colour indicates detected copy number. Note, the tetraploid cell from FNE1_1 was removed and these data are in part reproduced in Fig. 5A.

B Genome-wide chromosomal copy number profiles in one representative FNE1 cell and two P1 cells harbouring aneuploidies. Green, red and purple boxes indicate no deviation from mode, a copy number increase compared with mode and a copy number loss compared with mode, respectively.

Disease Models & Mechanisms • Supplementary information

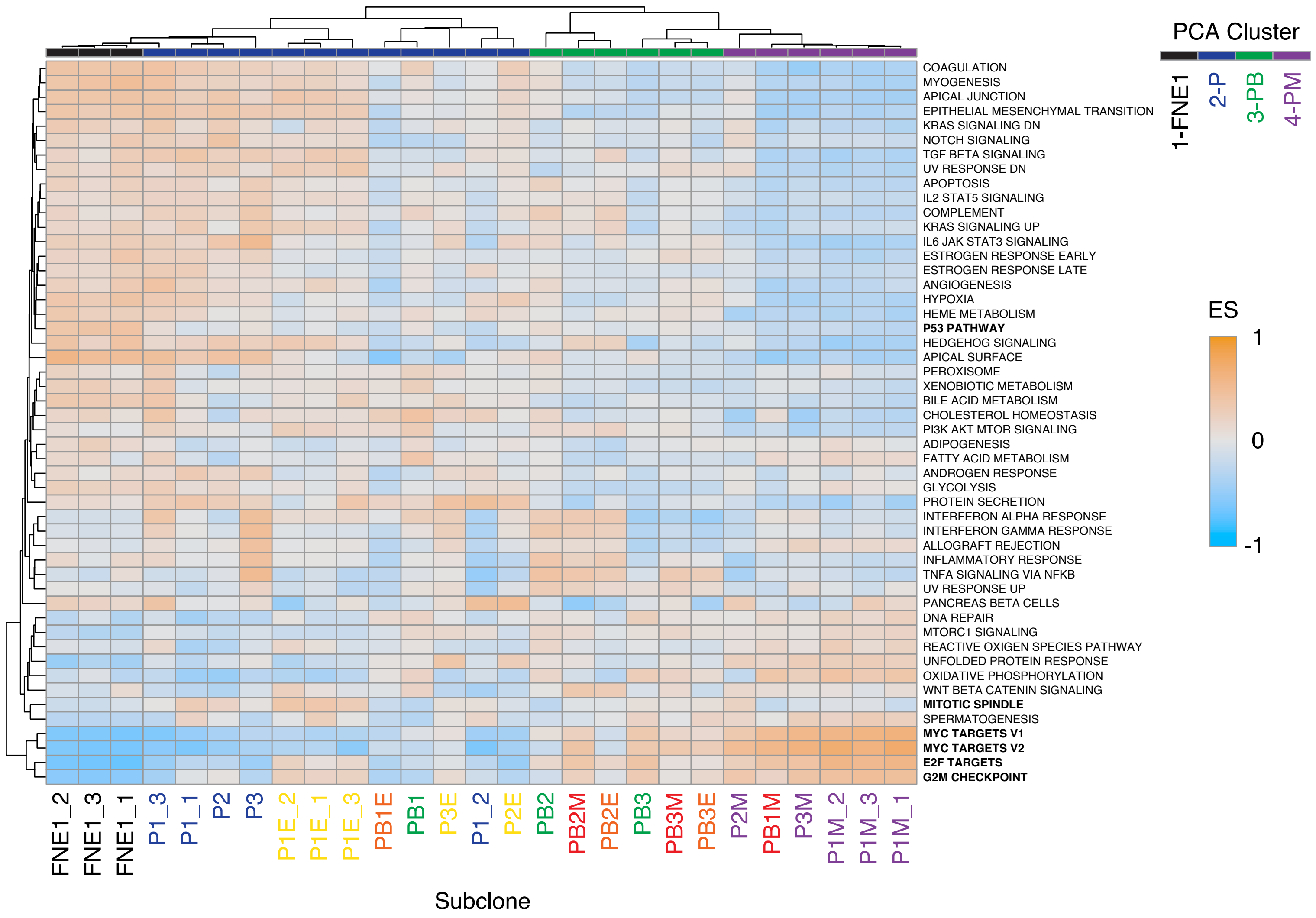

Fig. S6. Gene Set Variation Analysis Separates Parental and Mutant Samples Unsupervised hierarchical clustering of 27 cell lines based on enrichment scores calculated for Hallmark gene sets by gene set variation analysis (GSVA) from RNAseq. The top row indicates the PCA cluster of the respective sample, see Fig. 7A. Orange and blue shading indicate positive and negative enrichment scores, respectively.

Disease Models & Mechanisms • Supplementary information

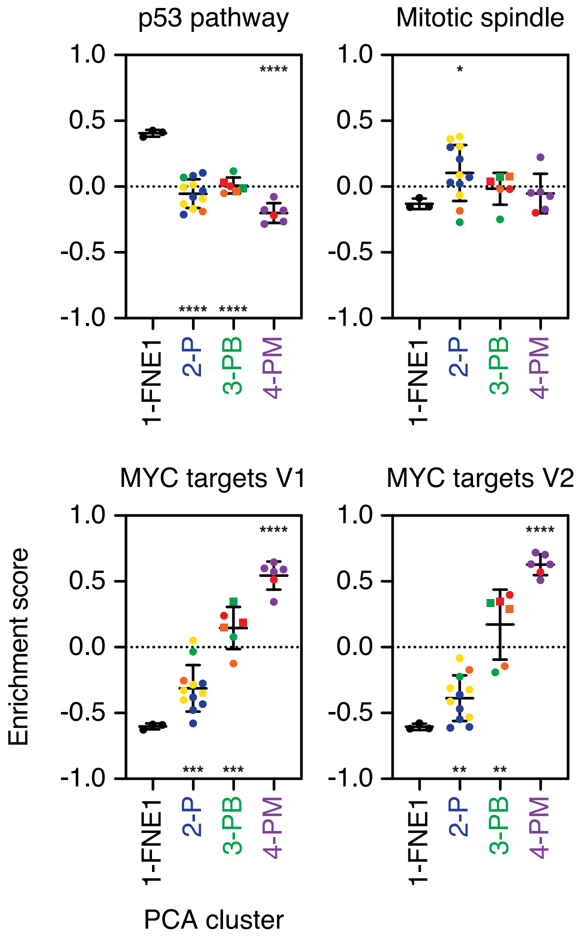

Fig. S7. Gene Set Variation Analysis Corroborates Genotypic Transcriptomic Features

Results from four representative Hallmark gene sets from Fig. 6B are shown. Samples were grouped based on PCA cluster allocation and the colour of individual data points corresponds to sample genotype as in Fig. 6A. For cluster 1 (FNE1): n=3 samples; cluster 2 (P): n=12; and clusters 3 and 4 (PB and PM): n=6. Note PB1 and PB1E/M samples are included in clusters 2 and 4, respectively, rather than 3 (see text). Samples from the PB3 lineage are depicted as squares. Horizontal bar and error bars indicate mean and standard deviation, respectively. Asterisks depict adj. p-value between indicated groups compared with cluster 1 (FNE1) by Brown-Forsythe and Welsh ANOVA where * adj. p-value ≤ 0.05, ** adj. p-value ≤ 0.005, *** adj. p-value ≤ 0.0005, **** adj. p-value < 0.0001. See Table S5.

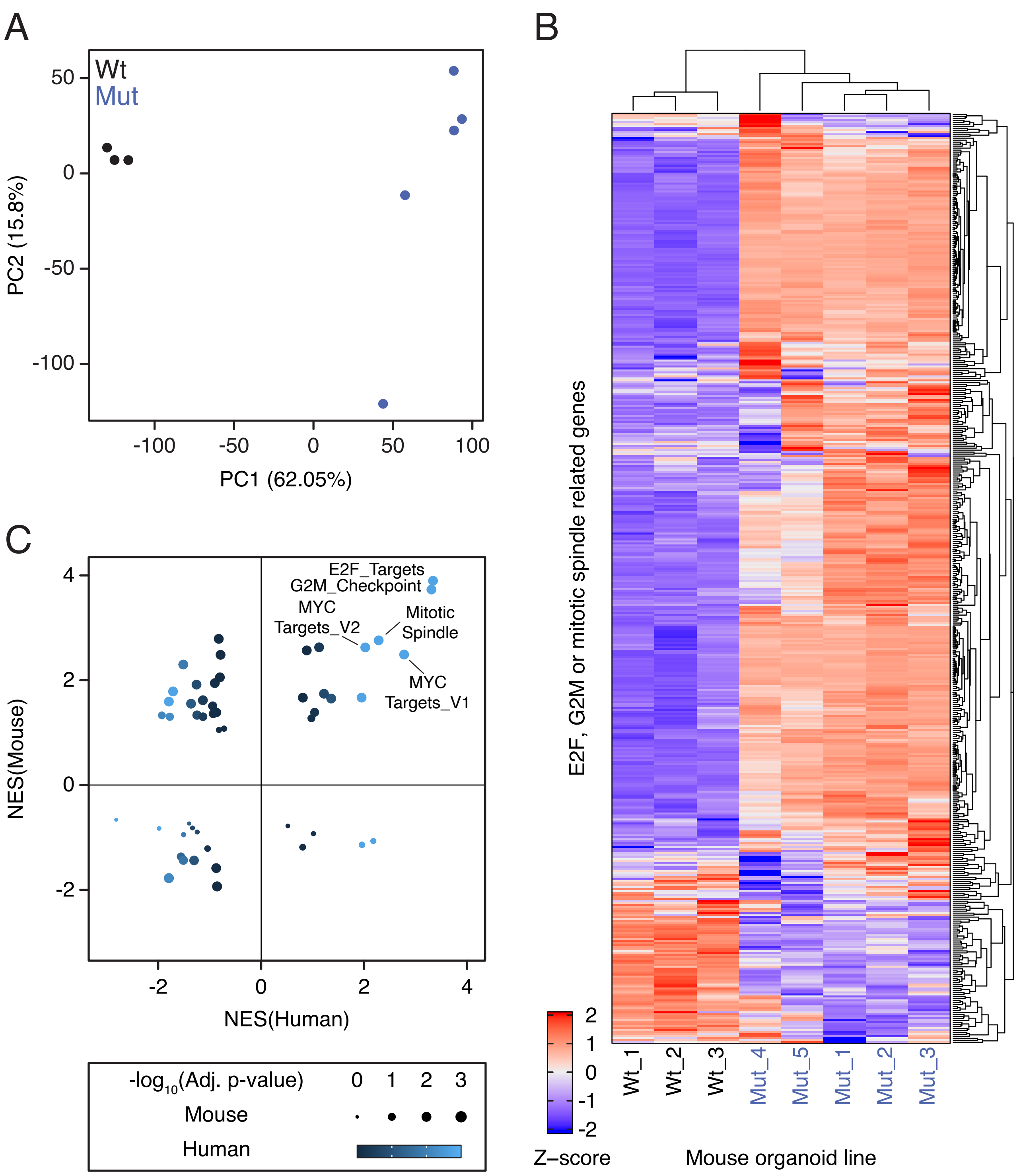

Disease Models & Mechanisms • Supplementary information

Fig. S8: Differential Expression of Cell Cycle Regulators in *TP53*-mutant Mouse Fallopian Tube Organoids Correlates with that of Human *TP53*-mutant Fallopian Tube-derived Subclones

A Principal component analysis (PCA) of publicly available RNA sequencing data from eight murine wildtype (Wt) and *Trp53*-mutant (Mut) organoids (Zhang et al., 2019). Percent variance of principle components 1 (PC1) and 2 (PC2) are indicated in parenthesis along axes. See also Table S6.

B Unsupervised hierarchical clustering based on the expression of 468 cell cycle regulators in the eight available mouse organoid samples. See also Table S7.

C Correlation of positively and negatively enriched gene sets when *TP53* is mutated in our human FNE1 model and the *Trp53*-mutant mouse organoids versus corresponding control cells. The size and the colour of the bubbles indicate significance in the mouse and human contrasts with wildtype, respectively. NES=normalized enrichment score.

Table S1. Summary of reagents and critical commercial kits, experimental models and software

Click here to download Table S1

Table S2. Summary of oligonucleotides used in this study (blue font indicates gRNA sequence)

Click here to download Table S2

Table S3. Filtered, quantile normalized, batch corrected, Log2 transformed RNA sequencing reads for cell line samples used as basis for all human RNA sequencing analyses downstream used to generate Fig. 6

Click here to download Table S3

Table S4. Mean enrichment scores for Hallmark gene sets calculated by gene set variation analysis of parental FNE1, P, PB and PM samples used to generate Fig. 6B

Click here to download Table S4

Disease Models & Mechanisms • Supplementary information

Table S5. Enrichment scores calculated in gene set variation analysis (GSVA) of all samples used to generate data in Table S4, Fig. 6B,C, S6, S7

Click here to download Table S5

Table S6. Filtered, quantile normalized, batch corrected, Log2 transformed RNA sequencing reads for organoid samples used as basis for all mouse RNA sequencing analyses downstream used to generate Fig. S8

Click here to download Table S6

Table S7. Z-scores calculated sample-wise for mouse organoid samples used to generate Fig. S8B

Click here to download Table S7

Disease Models & Mechanisms • Supplementary information
